# Engineered small extracellular vesicles loaded with miR-654-5p promote ferroptosis by targeting HSPB1 to alleviate sorafenib resistance in hepatocellular carcinoma

**DOI:** 10.1038/s41420-023-01660-2

**Published:** 2023-09-30

**Authors:** Jiao Sun, Qi Liu, Yanfang Jiang, Zhihui Cai, Hui Liu, Huaiwen Zuo

**Affiliations:** 1 Department of Gastroenterology, Shandong Provincial hospital affiliated to Shandong First Medical University, Jinan, China; 2 Department of Gastroenterology, Affiliated Qingdao Central Hospital of Qingdao University, Qingdao Cancer Hospital, Qingdao, China; 3 Aksu Vocational and Technical College School of Medicine, Aksu, China

**Keywords:** Cell death, Cancer

## Abstract

Sorafenib (sora) is the initial therapy for patients with progressive hepatocellular carcinoma (HCC), but the emergence of drug resistance has seriously impacted its therapeutic efficacy. However, the mechanism of sora resistance remains unclear, and effective strategies to overcome drug resistance are still lacking. By establishing a sora-resistant hepatocellular carcinoma cell line, we found that Heat Shock Protein Family B (small) Member 1 (HSPB1) was markedly upregulated in sora-resistant HCC cells. Further research revealed that the ferroptosis resistance induced by HSPB1 upregulation plays a crucial role in sora resistance. In addition, we confirmed that miR-654-5p enhances sora-induced ferroptosis by binding to HSPB1 and reducing its protein levels. To enhance miRNA stability and delivery efficiency in vivo, we used small extracellular vesicles (sEV) derived from human adipose mesenchymal stem cells as miR-654-5p carriers, creating engineered sEV (m654-sEV). The research demonstrated that m654-sEV effectively delivers miR-654-5p to HCC cells, targeting HSPB1 and enhancing sora-induced ferroptosis. This improves therapeutic effects on sora-resistant HCC cells and xenograft tumors, restoring their sensitivity to sora. In summary, m654-sEV, which targets HSPB1 via miR-654-5p delivery, represents a promising strategy for addressing sora-resistant issue. The combined use of m654-sEV and sora has the potential to significantly enhance therapeutic efficacy for patients with sora-resistant HCC.

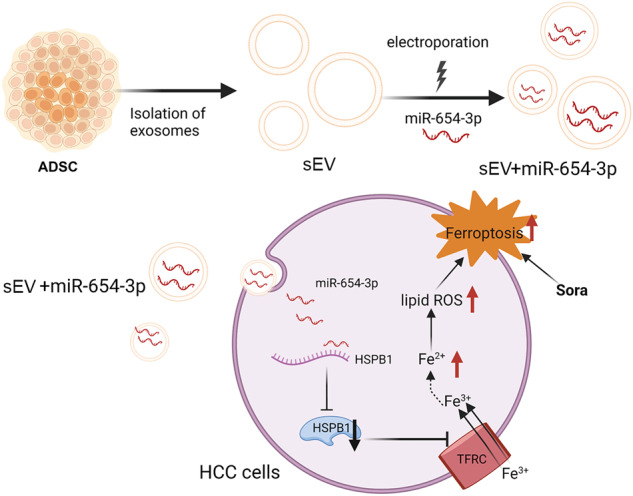

## Introduction

Hepatocellular carcinoma (HCC) is among the most widespread and fatal malignancies globally [1]. According to the WHO data, in 2020, there were 900,000 newly diagnosed cases of HCC and 830,000 associated deaths [2]. HCC is often diagnosed at advanced stages due to its ability to go undetected, and its insensitivity to chemotherapy poses significant treatment challenges [3]. Consequently, the pursuit of novel therapeutic approaches has become a priority in HCC research.

Sorafenib (sora), a multi-target kinase inhibitor, works by suppressing tumor cell proliferation and angiogenesis through multiple pathways [4]. Due to its remarkable effects, it has become the primary medication for advanced HCC [5]. Nevertheless, as its usage expands in clinical settings, acquired resistance to sora has gradually increased, and the specific mechanisms remain unclear, restricting its utility in HCC treatment [6]. Recent studies have found that sora’s efficacy against tumors partly dependent on its ability to induce ferroptosis in HCC cells, and resistance to ferroptosis is closely related to sora resistance [7]. Therefore, modulating ferroptosis-related molecules in HCC cells may provide new therapeutic strategies to counter sora resistance.

MicroRNA (miRNA) represents a type of endogenous non-coding RNA that degrade or inhibit the translation of target mRNAs by binding to their 3’UTR regions [8]. Their involvement in the biological behavior of tumor, including proliferation and invasion, is widely recognized [9, 10]. Importantly, miRNAs have shown the ability to suppress tumors in vitro tumor models [11]. Nevertheless, the systemic in vivo application of miRNAs faces substantial challenges for clinical use due to poor tumor targeting and vulnerability to degradation by endogenous RNases [12]. Hence, identifying safe and effective miRNA delivery vehicles is a critical step toward clinical applications.

Small extracellular vesicles (sEV) are cell-secreted natural vesicles ranging from 30 to 200 nm in diameter, featuring a bilayer membrane structure. This structure safeguards the enclosed miRNAs, proteins, and mRNAs from degradation by RNase [13]. sEV represent promising drug delivery vehicles, as they can be loaded with nucleic acids or drugs via direct incubation, electroporation, saponin, or freeze-thaw methods while exhibiting low immunogenicity and biotoxicity [14]. Mesenchymal stem cell-derived sEVs inherit the characteristics of their parent cells, have low immunogenicity and tumor-homing properties, and can effectively deliver payloads to tumor tissues, making them ideal miRNA carriers [15].

This work aims to investigate the association among ferroptosis resistance and sora resistance development in HCC cells, and to develop engineered sEV loaded with miR-654-5p (m654-sEV). The study will assess the therapeutic potential of m654-sEV in treating sora-resistant HCC, aiming to offer innovative insights and strategies for addressing sora resistance within the context of hepatocellular carcinoma.

## Results

### Establishment of HCC-R cell lines

**Fig. 1:**
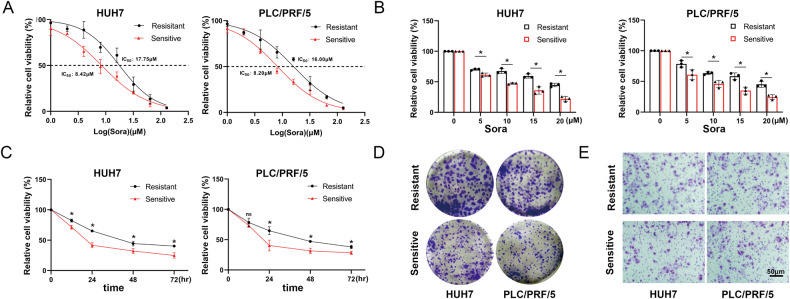
Establishment of HCC-R cell lines. **A** IC50 values of sora-sensitive and HCC-R cells upon treatment with sora. **B** Cell viability of sensitive and resistant cells treated with different concentrations of sora for 24 h. **C** Cell viability after treatment with 10 μM sora for different time periods. **D** Colony formation assay of sensitive and resistant cells under 1 μM sora treatment. **E** Transwell assay of sensitive and resistant cells under 1 μM sora treatment. *n* = 3 per group. Data are shown as the means ± SD, *p < 0.05. ns not significant. Sora: Sorafenib.

To investigate the potential mechanism underlying HCC resistance to sora, HCC-R cell lines (HUH7-R and PLC/PRF/5-R) were established by gradually increasing the sora concentration in the culture medium. CCK8 assays revealed that the IC50 values for HUH7-R and PLC/PRF/5-R were approximately two-fold higher than those for sensitive cells (Fig. 1A). Further cell viability assays demonstrated that HUH7-R and PLC/PRF/5-R exhibited reduced sensitivity to sora at various concentrations and time points (Fig. 1B). In addition, it was found that HUH7-R and PLC/PRF/5-R displayed enhanced proliferation (Fig. 1D) and migration (Fig. 1C) abilities compared to sensitive cells when treated with 1 μM sora.

### HSPB1 upregulation in HCC-R cell lines

**Fig. 2:**
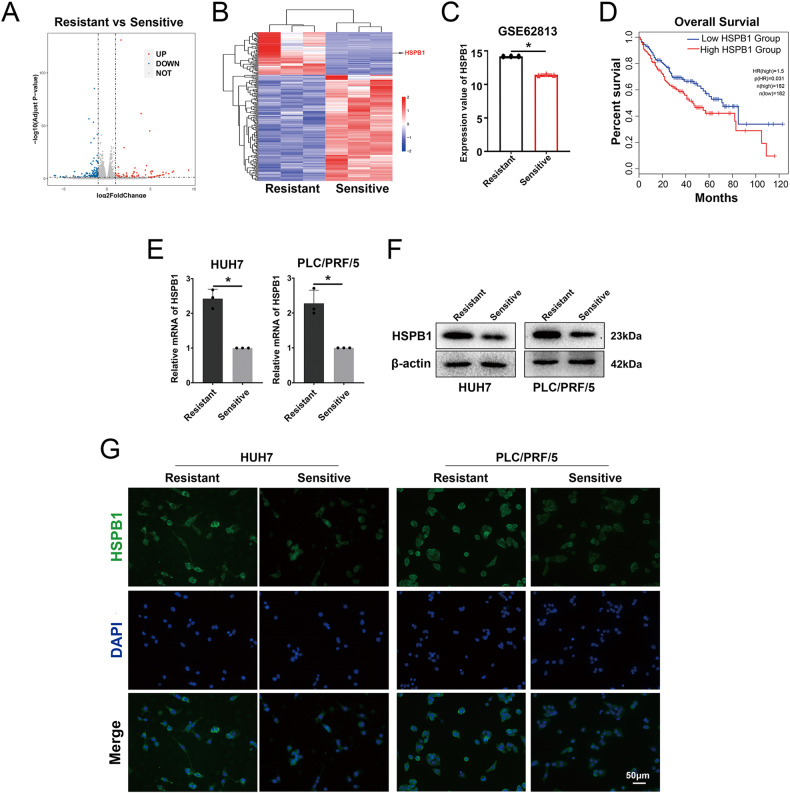
Upregulation of HSPB1 in HCC-R cells. **A** Volcano plot showing differentially expressed genes (DEGs) between resistant and sensitive cells with log2(fold change)>1. **B** Heatmap displaying the upregulation of ferroptosis-related genes HSPB1 in resistant cells. **C** HSPB1 mRNA levels in sora-resistant and sensitive cell lines from the GEO database (GSE62813). **D** Kaplan–Meier plots of overall survival for 364 HCC patients grouped by HSPB1 mRNA levels from the GEPIA dataset. **E**, **F** HSPB1 mRNA (**E**) and protein levels (**F**) in two types of cells. **G** Representative IF images of HSPB1 in in two types of cells. *n* = 3 per group. Data are shown as the means ± SD, *p < 0.05. ns not significant.

Recent studies have highlighted ferroptosis resistance as a key factor contributing to sora resistance. To investigate the possible association between sora resistance and ferroptosis, RNA sequencing was performed on HUH7-resistant and sensitive cells. A volcano plot illustrated the differentially expressed genes (DEGs) among the two cell types (Fig. 2A). The DEGs revealed a significant upregulation of the ferroptosis-negative regulator HSPB1 in sora-resistant cells (Fig. 2B). To further confirm HSPB1’s role in sora resistance, the GSE62813 transcriptome data from the GEO database was analyzed, and a significant upregulation of HSPB1 mRNA in HCC-R cells was found (Fig. 2C). Moreover, data from the GEPIA database [16] showed that HCC patients exhibiting elevated HSPB1 expression had shorter overall survival (Fig. 2D). These findings suggest a connection between HSPB1, HCC development, and sora resistance. Subsequently, significantly increased mRNA and protein expression of HSPB1 were observed in HUH7-R and PLC/PRF/5-R cells compared to sensitive cells (Fig. 2E–G).

### HSPB1 knockdown enhances ferroptosis in sora-treated resistant cells

**Fig. 3:**
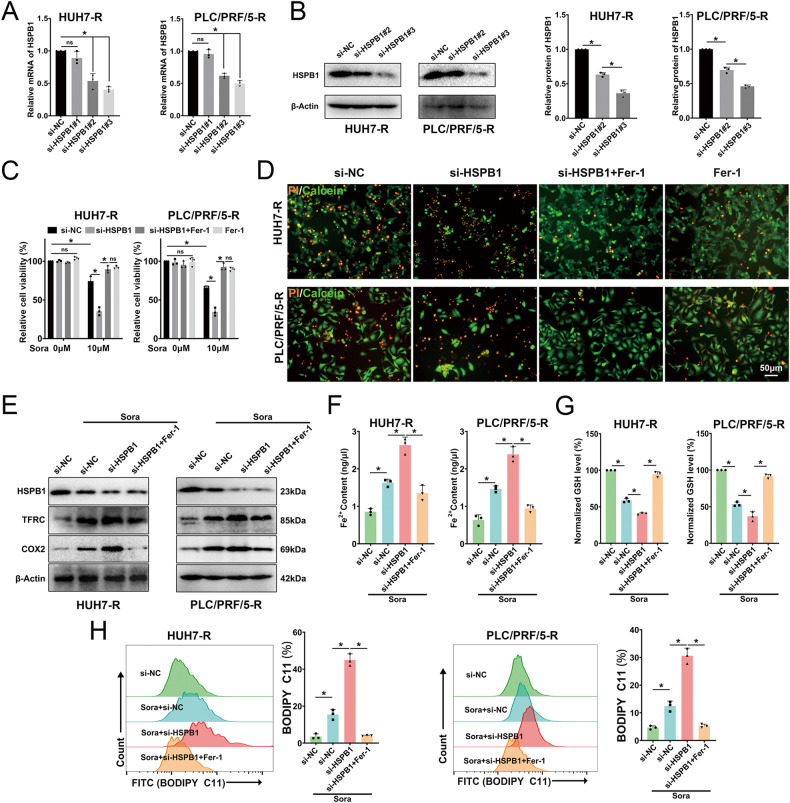
Knockdown of HSPB1 exacerbates sora-induced ferroptosis in HCC-R cell lines. **A**, **B** HSPB1 mRNA (**A**) and protein levels (**B**) after siRNA transfection. **C** Cell viability of cells exposed to DMSO or sora (10 μM for 24 h) combined with si-NC, si-HSPB1, si-HSPB1+Fer-1 (1 μM for 24 h), or Fer-1. **D** Representative images of PI/Calcein staining (red, dead cells; green, live cells). **E** Western blotting analysis of HSPB1, TFRC, and COX2 protein levels, with β-Actin as an internal reference. **F**, **G** Fe2+ content (**F**) and GSH level (**G**). **H** Lipid ROS measurements were performed via flow cytometry employing BODIPY C11. *n* = 3 per group. Data are shown as the means ± SD, *p < 0.05. ns not significant.

To examine the function of HSPB1 regarding sora resistance and its relationship with ferroptosis, siRNA was used to knock down HSPB1 in HUH7-R and PLC/PRF/5-R cells. The results demonstrated that si-HSPB1#3 significantly reduced HSPB1 mRNA and protein levels (Fig. 3A, B), and was used for subsequent experiments. HSPB1-knockdown HUH7-R and PLC/PRF/5-R cells were then treated with sora. The findings showed that HSPB1 knockdown reduced cell viability, while the ferroptosis inhibitor Fer-1 reversed the effect of HSPB1 knockdown (Fig. 3C, D). After treating cells with Deferoxamine (DFO, a chelating agent for iron), Z-VAD-FMK (ZVAD, an apoptosis inhibitor), Bafilomycin (BAF-A1, an autophagy inhibitor), and Necrosulfonamide (NSA, a necroptosis inhibitor), it was found that another ferroptosis inhibitor, DFO, could reverse the effect of HSPB1 knockdown, while the other inhibitors could not (Fig. S1). Previous research has shown that HSPB1 can inhibit ferroptosis by suppressing iron uptake via TFRC [17]. Western blot analysis was employed to further assess the expression of HSPB1, TFRC, and COX2 (a ferroptosis marker protein) (Fig. 3G). The results indicated that HSPB1 knockdown increased the protein content of TFRC in resistant cells, which was accompanied by increased COX2 and Fe2+ levels (Fig. 3H), decreased GSH (Fig. 3I), and increased lipid ROS levels (Fig. 3J). These findings suggest that HSPB1 knockdown exacerbates sora-induced ferroptosis in resistant cells by upregulating TFRC and Fe^2+^ levels.

### Overexpression of HSPB1 alleviates sora-induced ferroptosis in HCC-R cells

To further verify the role of HSPB1, we overexpressed HSPB1 (HSPB1-OE) in resistant HCC cells. The results revealed a considerable elevation in both mRNA and protein expression levels in HSPB1-OE cells (Fig. S2A, B). Further investigation showed that HSPB1-OE reversed the reduction in cell viability induced by sora, decreased TFRC and COX2 protein levels, and was followed by a reduction in Fe^2+^ (Fig. S2E), a rise in GSH (Fig. S2F), and a decrease in lipid ROS levels (Fig. S2G). These findings suggest that HSPB1 overexpression could inhibit sora-induced ferroptosis and reduce the therapeutic effect of sora on HCC cells.

### mir-654-5p targets HSPB1 to exacerbate ferroptosis in sora-treated resistant cells

**Fig. 4:**
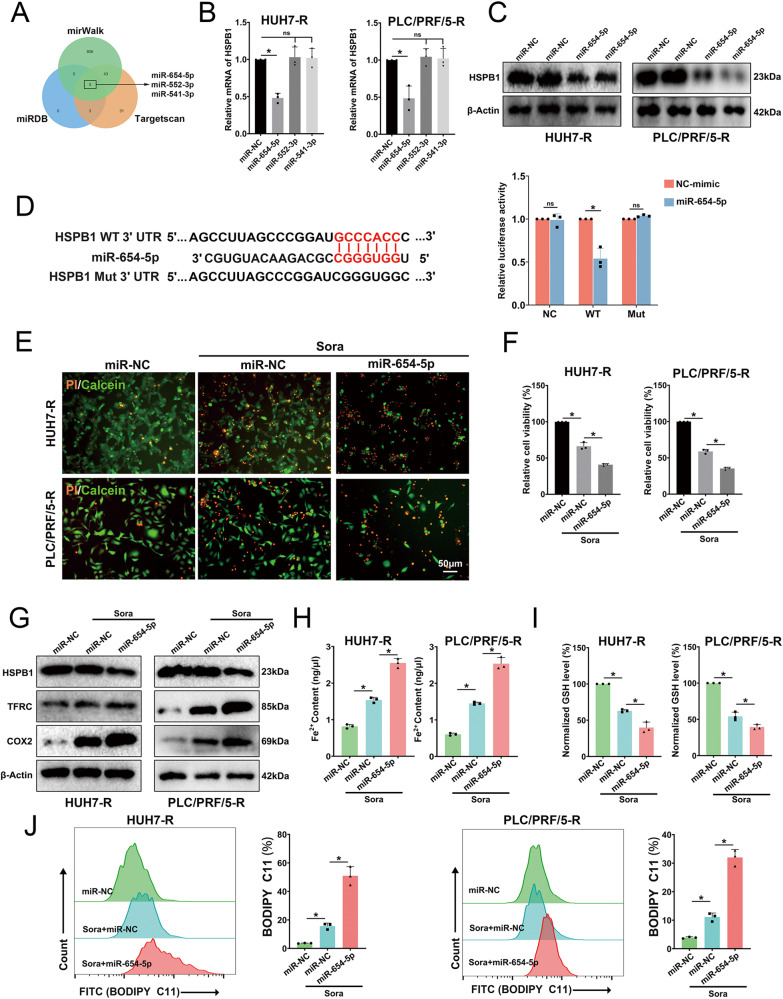
miR-654-5p targeting HSPB1 enhances sora-induced ferroptosis in HCC-R cells. **A** Venn diagram showing predicted targeted miRNAs of HSPB1 by miRwalk, miRDB, and TargetScan. **B** HSPB1 mRNA levels after transfection with miRNA mimic of miR-654-5p, miR-552-3p, and miR-541-5p. **C** HSPB1 protein levels after transfection with miR-654-5p. **D** Binding sites between miR-654-5p and HSPB1 (left) and dual-luciferase reporter assay (right). **E** Representative images of PI/Calcein staining (red, dead cells; green, live cells) f subsequent to cellular transfection using miR-654-5p and exposure to sora (10 μM for 24 h) either individually or in combination. **F** cell viability. **G**, **H** Fe^2+^ content (**G**) and GSH level (**H**). **I** HSPB1, TFRC, and COX2 protein levels in different groups, with β-Actin as an internal reference. **J** Lipid ROS levels measured by flow cytometry. *n* = 3 per group. Data are shown as the means ± SD, *p < 0.05. ns not significant.

MiRWalk, miRDB, and TargetScan online databases were used to predict miRNAs potentially targeting HSPB1. The prediction results indicated that miR-654-5p, miR-552-3p, and miR-541-3p could target HSPB1 (Fig. 4A). The three miRNA mimics (miR-654-5p, miR-552-3p, and miR-541-3p) were then transfected into resistant cells (Fig. S3A), and the results demonstrated that miR-654-5p reduced the mRNA and protein expression of HSPB1 (Fig. 4B, C). Subsequently, wild-type (WT) HSPB1 3’UTR and mutant (MT) reporter vectors containing the predicted binding sites were created and a dual-luciferase reporter experiment was carried out. The findings revealed that miR-654-5p markedly reduced luciferase activity of the WT group, but not the MT group (Fig. 4D). These findings confirm that miR-654-5p targets HSPB1. Importantly, transfection of miR-654-5p into cells intensified the cytotoxic effect of sora on HCC-R cells (Fig. 4E, F). Western blotting demonstrated that miR-654-5p suppressed HSPB1 expression, upregulated TFRC levels, and increased COX2 and Fe2+ (Fig. 4H), accompanied by a reduction of GSH and an elevation of lipid ROS levels (Fig. 4I, J).

**Fig. 5:**
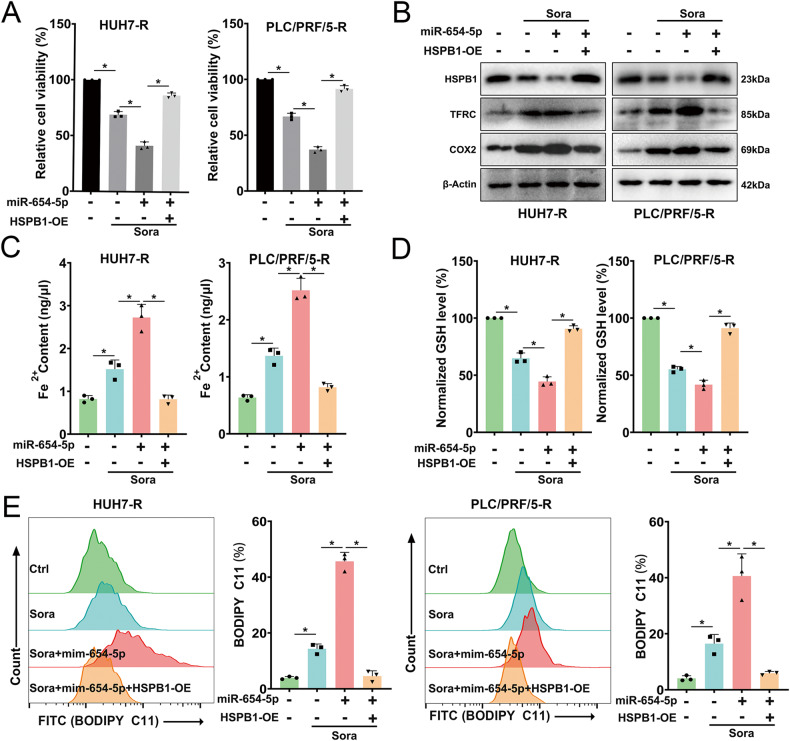
HSPB1 overexpression mitigates the pro-ferroptosis effect of miR-654-5p. **A** Cell viability after treating HCC-R Cells with Sora (10 μM for 24 h) after transfection with miR-654-5p solely or combined with HSPB1-OE. **B** HSPB1, TFRC, and COX2 protein levels, β-Actin as an internal reference. **C**, **D** Fe2+ contents (**C**) and GSH level (**D**). **E** Lipid ROS level assessed by flow cytometry. *n* = 3 per group. Data are shown as the means ± SD, *p < 0.05. ns not significant.

In order to clarify the roles of miR-654-5p and HSPB1 in HCC-R cells, HSPB1-OE plasmid and miR-654-5p were co-transfected into cells. The results demonstrated that HSPB1 overexpression could mitigate the enhanced killing effect of miR-654-5p on HCC cells induced by sora (Fig. 5A). Moreover, HSPB1-OE restored HSPB1 levels, reduced downstream TFRC levels, and decreased COX2 protein levels (Fig. 5B). In addition, overexpression of HSPB1 resulted in reduced Fe2+ and lipid ROS levels, while increasing GSH (Fig. 5C–E). Overall, miR-654-5p can target HSPB1 to enhance sora-induced ferroptosis in HCC-R cells.

### Preparation and characterization of engineered sEVs (m654-sEV)

**Fig. 6:**
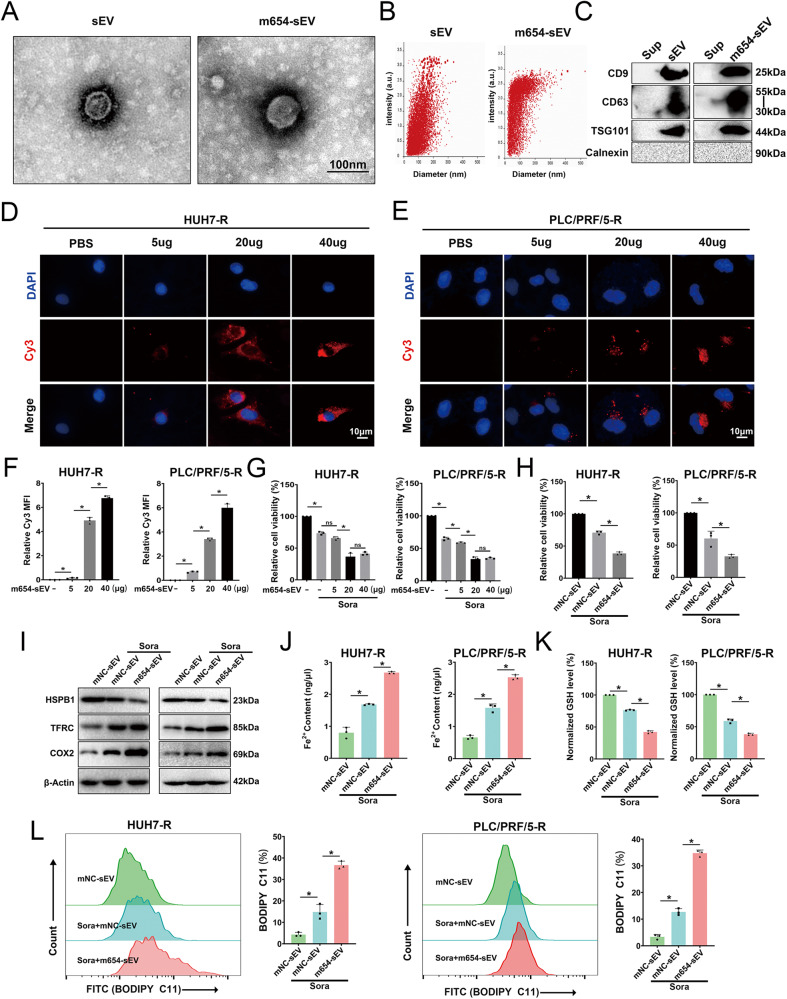
m654-sEV enhances sora-induced ferroptosis in HCC-R cells. **A**, **B** Morphology (**A**) and particle size distribution (**B**) of sEV and m654-sEV. **C** Marker proteins of Exo and m654-sEV. **D**–**F** Fluorescence images demonstrate the uptake of various concentrations of m654-sEV by HUH7-R (**D**) and PLC/PRF/5-R cells (**E**), with the uptake rate calculated based on the relative mean fluorescence intensity (MFI) of Cy3 (**F**). **G** Cell viability after 24 h of treatment with various concentrations of m654-sEV combined with Sora (10 μM). **H** Cell viability following 24 h treatment with different concentrations of m654-sEV combined with Sora (10 μM). **I**, **J** Fe2+ contents (**I**) and GSH level (**J**). **K** HSPB1, TFRC, and COX2 protein levels, with β-Actin as an internal reference. **L** Lipid ROS level assessed by flow cytometry. *n* = 3 per group. Data are shown as the means ± SD, *p < 0.05. ns not significant, sEV small extracellular vesicles, mNC-sEV miR-NC-loaded sEV. m654-sEV miR-654-5p-loaded sEV.

ADSCs exhibit a spindle-shaped appearance (Fig. S4A) and have the potential for osteogenic and adipogenic differentiation (Fig. S4B, C). Flow cytometry assessment indicated that ADSCs tested positive for CD29 and CD90, while being negative for CD45 and CD34 (Fig. S4D). Next, miR-654-5p was loaded into sEVs derived from ADSCs to construct engineered sEVs (m654-sEV). The qPCR showed a significantly elevated miR-654-5p concentration in m654-sEV compared to mNC-sEV (Fig. S3B). Transmission electron microscopy illustrated a comparable morphology between m654-sEV and sEV (Fig. 6A). Nanoparticle tracking analysis (NTA) revealed a similar particle size distribution for sEV and m654-sEV, mainly ranging from 50 to 150 nm (Fig. 6B). Both sEV and m654-sEV expressed surface marker proteins CD9, CD63, and TSG101, but not Calnexin (Fig. 6C).

### m654-sEV enhances sora-induced ferroptosis in drug-resistant HCC cells

Cy3-labeled miR-654-5p was prepared and used to test whether m654-sEV could be taken up by HUH7-R and PLC/PRF/5-R cells at different concentrations (Fig. 6D, E). The results indicated that the Cy3 fluorescence intensity inside HUH7-R and PLC/PRF/5-R cells increased with the concentration of m654-sEV, indicating that miR-654-5p could be delivered to HCC cells through m654-sEV (Fig. 6F). After treatment with different concentrations of m654-sEV, sora had an enhanced killing effect on cells, Nonetheless, the 20 μg/ml and 40 μg/ml concentrations did not exhibit any notable differences (Fig. 6G); Consequently, 20 μg/ml of sEVs were used for subsequent experiments. The results showed that m654-sEV significantly enhanced sora-induced cell death in drug-resistant HCC cells (Fig. 6H). Western blotting analysis demonstrated that m654-sEV could inhibit HSPB1 expression, increase TFRC and COX2 levels (Fig. 6I), and result in a rise in Fe2 + , a reduction in GSH, and an increase in lipid ROS levels (Fig. 6J–L). These findings indicate that m654-sEV can enhance ferroptosis and thereby increase the efficacy of sora against tumors, which can be attributed to the suppression of HSPB1 by miR-654-5p.

### m654-sEV enhances the therapeutic efficacy of sora in sora-resistant xenograft model

**Fig. 7:**
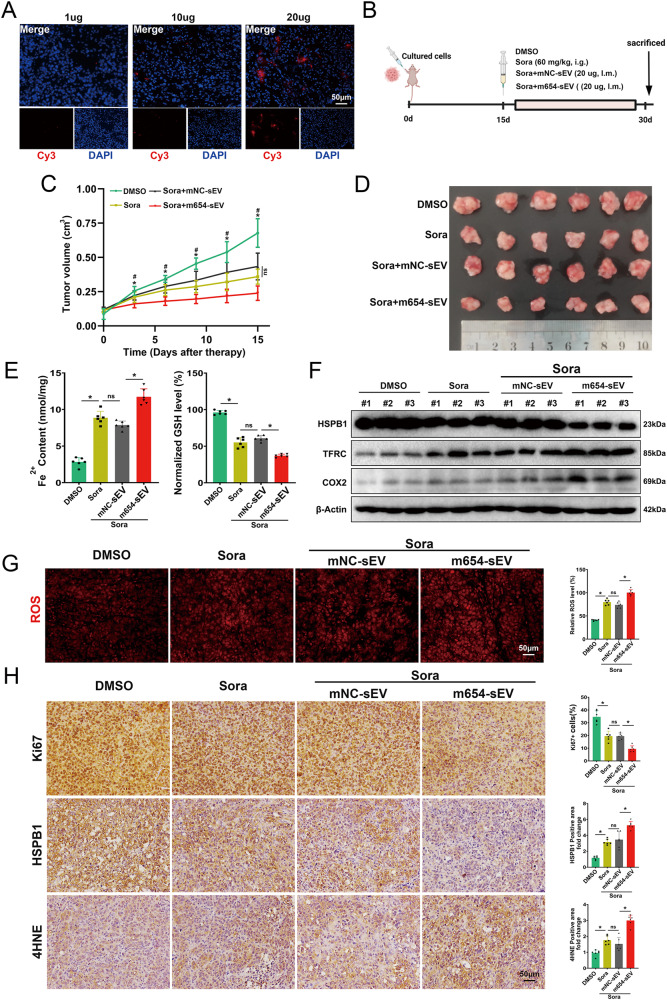
m654-sEV enhances sora-induced ferroptosis in vivo. **A** In vivo biodistribution of various concentrations of m654-sEV. **B** Graphical representation of the animal experiment design. On the 15th day after the injection of HCC-R cells, the mice were treated with DMSO, Sora, Sora+mNC-sEV, and Sora+m654-sEV, and were sacrificed on the 30th day. **C** Tumor volume changes in mice after treatment with DMSO, Sora, Sora+mNC-sEV, and Sora+m654-sEV, with volume recorded every 3 days (*n* = 6). **D** Tumor images of mice in each group. **E** Fe2+ contents (left) and GSH level (right) (*n* = 6). **F** HSPB1, TFRC, and COX2 protein levels in each group, with β-Actin as an internal reference (*n* = 3). **G** Representative DHE staining images reflecting the relative ROS content (*n* = 6). **H** Representative IHC images of Ki67, HSPB1, and 4HNE. Data are shown as the means ± SD, *p < 0.05. ns not significant.

To further appraise the therapeutic effect of m654-sEV combined with sora in sora-resistant HCC, subcutaneous xenograft tumor models were established. In vivo biodistribution experiments showed that m654-sEV was significantly enriched in xenograft tumor tissue after injection of 20 μg per mouse. (Fig. 7A). The experimental design and treatment regimen are shown in the schematic diagram in Fig. 7B. The growth curve and appearance of the tumors indicated that the combination of sora and m654-sEV considerably suppressed tumor growth in comparison to sora treatment alone (Fig. 7C, D).

To confirm the role of m654-sEV in modulating sora-induced ferroptosis in sora-resistant HCC. The ferroptosis-associated markers were detected in the xenografts. The results demonstrated that the combination of m654-sEV and sora treatment more effectively inhibited HSPB1 expression, increased levels of TFRC, COX2, Fe2 + , and ROS, and was accompanied by a decrease in GSH (Fig. 7E–G). These findings are highly consistent with the results of in vitro experiments. Furthermore, immunohistochemical staining suggested that positive reactions of HSPB1 and Ki67 in the combination treatment group were weaker than those in the sora-alone treatment group, accompanied by an increase in the positive reaction of 4-HNE (a ferroptosis marker). These findings indicate that targeting HSPB1 with m654-sEV to upregulate sora-induced ferroptosis can enhance the efficacy of sora and inhibit tumor growth.

## Discussion

The application of sora has substantially prolonged the survival time of patients with advanced HCC. However, the emergence of drug resistance has led to a significant decline in its therapeutic effects [18]. Therefore, elucidating the mechanism of sora resistance and identifying potential therapeutic strategies are of paramount importance. In the present study, we established HCC-R cells and found that the upregulation of the ferroptosis negative regulator HSPB1 led to ferroptosis resistance related to sora-resistance. Subsequently, it was demonstrated that miR-654-5p can target HSPB1. ADSCs-derived sEVs were then used as miR-654-5p delivery carriers, successfully delivering miR-654-5p to HCC-R cells. This approach restored the sensitivity of HCC-R cells to sora by promoting ferroptosis via the miR-654-5p/HSPB1 axis.

Ferroptosis is an iron-dependent, lipid peroxidation-mediated programmed cell death, serving as a crucial mechanism for sora to treat HCC [19]. Recent research has demonstrated that ferroptosis resistance is one of the key reasons for sora resistance [20]. Several key molecules, such as Metallothionein-1G, Glutathione S-transferase zeta 1, and SLC27A5, and Metallothionein-1G, have been demonstrated to be involved in ferroptosis-related Sorafenib resistance. However, the role of ferroptosis in Sorafenib resistance remains not fully elucidated [21–23]. In this study, HCC-R cells were established and exhibited significant upregulation of HSPB1. HSPB1 is a small heat shock protein closely associated with the growth and progression of various tumors, such as HCC, colorectal cancer, and glioma [24–26]. According to previous research, HSPB1 inhibits TFRC, which leads to a reduction in iron entry into cells. Consequently, this diminishes the labile iron pool (LIP) within cells and hampers ferroptosis [17]. In this study, we found that knocking down HSPB1 in HCC-R cells resulted in a rise in sora-induced cell death, accompanied by the upregulation of ferroptosis markers. Furthermore, ferroptosis inhibitors Fer-1 and DFO could reverse cell death caused by HSPB1 knockdown, while inhibitors of other cell death types were ineffective. In contrast, overexpressing HSPB1 could inhibit ferroptosis and diminish sora’s effectiveness. These outcomes suggest that the elevation of HSPB1-triggered ferroptosis resistance plays a crucial role in sora resistance. Mechanistically, knockdown of HSPB1 can increase TFRC levels and intracellular Fe2+ content, thereby enhancing ferroptosis, which is consistent with previous reports [17]. Previous studies have shown that miR-654-5p can modulate the malignant behavior of various cancer cells. In lung and colorectal cancers, miR-654-5p inhibits tumor cell proliferation, migration, and epithelial-mesenchymal transition [27, 28]. Similarly, miR-654-5p can target Epithelial Stromal Interaction 1 to weaken breast cancer cell proliferation and invasion while promoting cell apoptosis [29]. However, Zhou et al. [30] found that miR-654-5p can promote tumor progression by enhancing the malignant behavior of cells in gastric cancer. These contradictory results might be linked to variations in target genes among diverse cancer cells. In this work, we determined that miR-654-5p can target HSPB1 to enhance sora-induced ferroptosis, thereby improving sora’s therapeutic effect. It should be noted that miRNAs have extremely complex mechanisms of action, and a single miRNA can regulate the function of multiple mRNAs, which implies that miR-654-5p may also regulate other mRNAs [31]. Thus, by overexpressing HSPB1 to reverse the effects of miR-654-5p, we demonstrated that HSPB1 is the primary target of miR-654-5p.

In recent years, the therapeutic potential of miRNAs in cancer therapy has been demonstrated [32]. However, the in vivo application of miRNAs still faces significant challenges. First, miRNAs are easily degradable molecules and are difficult to maintain in a stable form in the physiological environment [33]. Although researchers have attempted to improve their stability through chemical modifications, such as adding methoxy groups or thio-skeletons, these modifications may alter the activity of miRNAs and increase their toxicity [34]. Secondly, after injection, miRNAs are widely distributed throughout the body and may have unintended effects on different cell types, leading to off-target effects [35]. Consequently, the development of secure and effective delivery systems is an essential aspect of in vivo applications.

sEV derived from mesenchymal stem cells have excellent biocompatibility and tumor-targeting properties, serving as efficient miRNA delivery carriers [36]. Lou et al. [37] demonstrated that sEVs derived from human ADSCs loaded with miR-199 enhanced the sensitivity of hepatocellular carcinoma (HCC) to doxorubicin. MiR-122-modified sEVs from ADSCs increased the chemosensitivity of HCC [38]. In this study, we successfully constructed engineered m654-sEV by loading miR-654-5p into ADSCs-derived sEVs through electroporation. In vitro and in vivo experiments showed that m654-sEV could deliver miR-654-5p to HCC cells and enhance sora-induced ferroptosis by regulating HSPB1, significantly inhibiting the growth of xenograft tumors. This suggests that the engineered m654-sEV can reduce the degree of sora resistance in HCC.

Currently, sEVs can be administered through various routes such as intravenous injection, intratumoral injection, and intraperitoneal injection [39]. sEV injected intravenously are rapidly distributed in the liver and spleen and remain there for an extended period but are difficult to accumulate in subcutaneous tumors. In contrast, direct intratumoral injection can effectively stabilize sEVs within tumor tissues for over 24 h [40]. Therefore, in our experiments, we used intratumoral injection for subcutaneous tumors, and the results showed that sEVs could still be detected in the tumor tissue three days after injection, proving the feasibility of this method. Due to the liver enrichment characteristics of sEVs, intravenous injection is not suitable for treating subcutaneous tumors in mice. However, for primary liver tumors, intravenous injection seems to be an efficient method.

There are certain limitations to our research. Firstly, due to the use of subcutaneous tumor models, we are unable to accurately assess the enrichment of m654-sEV in situ HCC. Secondly, although we confirmed that HSPB1 is the main target of miR-654-5p, considering the multiple targeting of miRNAs, it may still function by affecting other mRNAs. Lastly, we have not yet detected the expression of HSPB1 in Sora-resistant HCC. These aspects should be explored further in our future research.

In conclusion, our findings show that the increased resistance to ferroptosis due to HSPB1 upregulation plays a crucial role in sora resistance in HCC. Engineered m654-sEV can enhance sora-induced ferroptosis through the miR-654-5p/HSPB1 axis and shows potential for restoring sora sensitivity in sora-resistant HCC.

## Materials and methods

### Cell culture

The human HCC cell line HUH7 and PLC/PRF/5 were procured from the Shanghai Institute for Biological Science (Shanghai, China). HUH7 and PLC/PRF/5 were cultured in DMEM (Gibco, Carlsbad, CA, USA) and MEM (Gibco), respectively, with 10% FBS (Gibco) and 1% penicillin-streptomycin (Gibco) added. Human adipose mesenchymal stem cells (ADSCs) were obtained from Oril cells (HUXMD-01001, Guangzhou, China) and cultured in serum-free medium (HUXMD-90062, Oril cells).

### Establishment of HCC-R cell lines

HUH7 and PLC/PRF/5 cells were initially exposed to sora (0.5 μM). The concentration was then gradually increased in 1 μM increments, selecting for surviving cells at each step. Over 6 months, sora-resistant HCC cell lines (HCC-R) HUH7-R and PLC/PRF/5-R were successfully established. HUH7-R and PLC/PRF/5-R cells consistently cultured in medium containing sora (1 μM) to maintain resistance.

### Characterization and functional identification of ADSCs

ADSCs underwent staining with Alizarin Red S and Oil Red O after inducing differentiation using the Osteogenic Differentiation Kit and Lipogenic Differentiation Kit (Oril cell) following the manufacturer’s instructions. The fluorophore-conjugated antibodies CD29 (303004), CD90 (328108), CD34 (343504), and CD45 (982322), FITC isotype control (400107), and PE isotype control (400114) were employed to identify surface marker proteins of ADSCs using flow cytometry. These antibodies were obtained from BioLegend (San Diego, CA, USA).

### Isolation of sEVs

The cell supernatant of ADSCs was collected according to the previous sEV isolation method [41], with dead cells and cellular debris eliminated via centrifugation at 300 × *g* for 15 min, followed by 3000 × g for 25 min at 4 °C. The supernatant was then centrifuged at 10,000 × *g* for 25 min, filtered through a 0.22 μm filter, and centrifuged once more at 110,000 × g for 70 min at 4 °C. Protein concentrations were assessed using the BCA assay, while the sEV precipitate was dissolved in PBS and stored at −80 °C until required.

### Preparation and characterization of engineered m654-sEV

Electroporation mixtures were prepared by mixing sEVs at a concentration of 100 ug/ml with miR-654-5p or miR-NC at a 1:1 (wt/wt) ratio. Electroporation was performed using a CUY21 EDIT II (BEX, Japan) under the following conditions: 110 V, 6 ms pulse/10 ms pause, 10 cycles, capacitance of 940μF. Samples were subsequently collected and incubated at 37 °C for 30 min, followed by centrifugation at 110,000 × *g* for 70 min at 4 °C to remove the unloaded miRNA. Nanoparticle tracking analysis (NTA) was performed to identify m654-sEV morphology and particle size distribution. Western blot analysis served to identify the sEV protein markers, and qRT-PCR was conducted for assessing miR-654-5p levels within sEV.

### Cellular uptake of m654-sEV

The Cy3-labeled miR-654-5p was incorporated into sEVs using the electroporation method described earlier and then co-cultured with HUH7-R or PLC/PRF/5-R cells in the medium for 24 h. Subsequently, the cells underwent three PBS washes, followed by DAPI staining of the nuclei. The cellular uptake of sEVs was assessed with a fluorescence microscopy (Nikon, Tokyo, Japan).

### In vivo biodistribution of m654-sEV

sEV carrying Cy3-labeled miR-654-5p were injected into tumor at different concentrations, and frozen sections of tumor tissues were taken 3 days later and observed under a fluorescence microscope after DAPI-stained nuclei.

### Reagents

Erastin (E7781), Ferrostatin-1 (SML0583), Deferoxamine (D9533), and Necrosulfonamide (480073) were obtained from Merck KGaA (Darmstadt, Germany). Bafilomycin A1 (HY-100558), sorafenib (HY-10201), and ZVAD-FMK (HY-16658B) were obtained from Med Chem Express (MCE, Shanghai, China).

### Colony formation assay

After cell digestion, cells were counted, and 500 cells being seeded in six-well plates containing 1 μM sora, with the medium being replaced at 3-day intervals. The cell supernatant was discarded after 2 weeks, and 4% paraformaldehyde fixation was followed by 0.5% crystalline violet staining. Colonies were assessed using a microscope (Nikon), and cell numbers greater than 50 were recorded as one colony.

### Transwell assay

Briefly, 1 × 10^5 cells were seeded in the upper chamber of a Transwell insert (Corning Inc., Corning, NY). Prepare the medium by adding 10% Fetal Bovine Serum (FBS) in the upper chamber and 20% FBS in the lower chamber. Following a 24 h period, the Transwell insert was removed, while the remaining cells in the top compartment were cleared. Fix the migrated cells on the membrane’s bottom surface using 4% paraformaldehyde, then apply 0.5% crystal violet stain. Lastly, observe the migrated cells using a light microscope.

### Cell viability assay

Briefly, 5000 cells were seeded in a 96-well plate format and exposed to the relevant drug treatments. The cell counting kit-8 solution (CCK-8, from Beyotime, Shanghai, China) was then introduced into every well and allowed to incubate for 2 h at 37 °C. Absorbance measurements at 450 nm were taken using an absorbance microplate reader (Dojindo, Kumamoto, Japan).

### Transfection

HSPB1’s coding sequence was integrated into the vector pcDNA3.1 (Genepharma, China) for creating an HSPB1 overexpression plasmid. siRNA, siRNA-negative control (NC), Cy3-labeled miR-654-5p-mimic, and miR-NC were obtained from Genepharma. Lipofectamine 3000 reagent (Invitrogen, USA) was employed for transfections. Cells (2 × 10^5) were cultured in 6-well plates at around 60% confluence before transfection. Target gene expression was assessed after 72 h.

### PI/Calcein staining

After the cells underwent the corresponding drug treatment, the culture medium was gently removed. The PI/Calcein working solution (C2015L, Beyotime) was incubated with cells for 30 min. A fluorescence microscope was employed observe cells, where red fluorescence signified dead cells and green fluorescence denoted living cells.

### Western blotting

Cell and tissue lysis was performed with RIPA buffer (P0013E, Beyotime) to for protein extraction. Protein concentrations were measured utilizing a BCA protein assay kit (Solarbio, Beijing, China). The Western blot procedure was executed based on a previously described method [41]. Primary antibodies, such as HSPB1 (1:1000, sc-13132), COX2 (1:500, sc-376861), and β-actin (1:1000, sc-47778), were acquired from Santa Cruz Biotechnology (Santa Cruz, CA). TSG101 (1:2000, ab125011), CD63 (1:2000, ab217345), CD9 (1:2000, ab307085), calnexin (1:2000, ab10286), and TFRC (1:2000, ab214039) were obtained from Abcam (Abcam, Cambridge, MA, USA).

### Quantitative real-time PCR (qRT-PCR)

Total RNA was isolated from tissues or cell specimens employing Trizol reagent (Thermo Fisher Scientific) per the provided guidelines. cDNA was synthesized using the PrimeScript RT reagent Kit (Takara, Kyoto, Japan). β-Actin and U6 served as control references for mRNA and miRNA accordingly. The 2^−ΔΔCt^ approach was determine fold changes. Reverse transcription and amplification primers are listed in Supplementary Table 1.

### RNA sequencing

Total RNA was isolated from HUH7-R cells (*n* = 3) and sensitive cells (*n* = 3) using TRIzol. RIN values for RNA were ascertained using an Agilent 4150 Bioanalyzer (Agilent Technologies, CA, USA). cDNA was synthesized using RNase H and DNA polymerase I as templates, and the cDNA library was constructed via PCR. Sequencing was performed on the Illumina 2000 system. The raw sequencing information was submitted to the NCBI SRA under BioProject: PRJNA963190.

### Immunofluorescence and immunohistochemistry

Logarithmically growing cells were seeded onto cell slides. After the corresponding treatments, slides were removed, fixed, and blocked. Then, the appropriate primary antibody was applied and left to incubate at 4 °C for the night. The following day, corresponding secondary antibodies were applied, nuclei were stained with DAPI, and samples were observed under a fluorescence microscope.

In the immunohistochemistry procedure, sections embedded in paraffin underwent deparaffinization and rehydration, followed by antigen retrieval, blocking, and exposure to primary antibodies at 4 °C overnight. The next day, sections were incubated with HRP-labeled secondary antibodies for 30 min, and subsequently underwent DAB development. Nuclei were stained with hematoxylin, and sections were dehydrated, cleared, and mounted before being observed under a microscope. Primary antibodies used included HSPB1 (1:200, Santa Cruz), 4HNE (1:50, ab48506, Abcam), and Ki67 (1:200, sc-23900, Santa Cruz).

### Dual luciferase reporter gene assay

HSPB1 3′UTR sequence capable of binding to miR-654-5p and its mutant sequence were integrated into the pmirGLO luciferase reporting vector to create HSPB1-3′UTR (WT) and HSPB1-3′UTR (MUT) constructs respectively. miR-654-5p/NC was co-transfected with WT/MUT plasmids into HEK-293T cells. Following a 48 h incubation period, luciferase activity was assessed, normalizing firefly luciferase activity to Renilla luciferase activity.

### Lipid reactive oxygen species (ROS) assay

The BODIPY 581/591 C11 (Thermo Fisher Scientific, MA, USA, D3861) probe was utilized to measure intracellular lipid ROS levels. Cells were cultured in a 12-well plate, and after respective treatments, the cell supernatant was removed. Add the working solution prepared from serum-free medium (5 μM) and incubate at 37 °C in the dark for 20 min. Cells were then gathered, washed three times with PBS, and lipid ROS levels were assessed using flow cytometry.

### Fe2+ and GSH assay

The Fe2+ assay kit (MAK025, Merck) and the GSH assay kit (S0053, Beyotime) were employed to measure Fe2+ and GSH levels in cells and tissues, following the guidelines provided by the manufacturers. The absorbance of samples treated with the kits was detected at 593 nm (Fe2 + ) and 412 nm (GSH), and their contents were calculated.

### ROS assay

Frozen tissue sections were stained with 10 μM dihydroethidium (MCE, HY-D0079) for 30 min, followed by observation under a fluorescence microscope. Fluorescence intensity was quantified using Image J software [42].

### Animal experiment

Six-week-old female BALB/c nude mice were procured from Vital River (Beijing, China), which have free access to water and food in a specific pathogen-free (SPF) environment. Xenograft tumor models were established by administering 2 × 10^6 HUH7-R cells into the right axillary region of the mice. Tumor size was determined employing the equation: 0.5 × (L × W^2) (with L denoting length and W signifying width. When the tumor volume reached 0.1 cm^3^, mice were randomly assigned to four treatment groups: DMSO group (intragastric administration of DMSO); sora group, (intragastric administration of sora at a dose of 60 mg/kg every 2 days, as previously reported [43]); sora+ mNC-sEV group (sora combined with intratumoral injections of mNC-sEV every 3 days); sora+m654-sEV group (sora combined with intratumoral injections of miR-654-sEV (20 μg total protein in 30 μL PBS) every 3 days). Tumor size were measured every 3 days, and after 15 days, mice were euthanized, and tumor tissues were collected for subsequent assays.

All animal experiments were conducted in accordance with the ARRIVE guidelines and were approved by the Institutional Animal Care and Use Committee of Shandong University.

### Statistical analysis

GraphPad Prism 7.0 (San Diego, CA) was employed for data presentation and analysis. At least three independent replicates were performed for each experiment. Data were presented as mean ± standard deviation, with Student’s *t* test comparing two groups. One-way analysis of variance was used for comparisons among multiple groups. A *p* value < 0.05 was considered statistically significant.

### Supplementary information

Supplementary tables

Supplementary figure and figure legends

Original western blots

## Data Availability

Data supporting the results of this study are available on request.
