# Supplementary material for: Engineered small extracellular vesicles loaded with miR-654-5p promote ferroptosis by targeting HSPB1 to alleviate sorafenib resistance in hepatocellular carcinoma
Source: Cell Death Discov. 2023 Sep 30;9:362. doi: 10.1038/s41420-023-01660-2 (PMC10542782; doi:10.1038/s41420-023-01660-2)
Supplement: Supplementary file 1 — Supplementary tables [file 41420_2023_1660_MOESM1_ESM.docx]

**Supplementary Table 1 Primer sequences for qRT-PCR**

| Gene name |  | Sequence (5’-3’) |
| --- | --- | --- |
| β-actin | FORWARD | CACCATTGGCAATGAGCGGTTC |
|  | REVERSE | AGGTCTTTGCGGATGTCCACGT |
| HSPB1 | FORWARD | CTGACGGTCAAGACCAAGGATG |
|  | REVERSE | GTGTATTTCCGCGTGAAGCACC |
| hsa-miR-654-5p | FORWARD | AACAATTGGTGGGCCGCAGAA |
|  | REVERSE | ATCCAGTGCAGGGTCCGAGG |
|  | RT Primer | GTCGTATCCAGTGCAGGGTCCGAGGTATTCGCACTGGATACGACGCACAT |
| hsa-miR-541-3p | FORWARD | AAGGTTGTTGGTGGGCACAGAA |
|  | REVERSE | ATCCAGTGCAGGGTCCGAGG |
|  | RT Primer | GTCGTATCCAGTGCAGGGTCCGAGGTATTCGCACTGGATACGACAGTCCA |
| hsa-miR-552-3p | FORWARD | GACCGCAAACAGGTGACTGGT |
|  | REVERSE | ATCCAGTGCAGGGTCCGAGG |
|  | RT Primer | GTCGTATCCAGTGCAGGGTCCGAGGTATTCGCACTGGATACGACTTGTCT |
| U6 | FORWARD | CTCGCTTCGGCAGCACAT |
|  | REVERSE | TTTGCGTGTCATCCTTGCG |

**Supplementary Table 2 miRNA mimic and siRNA sequence.**

| miRNA mimic/siRNA |  | Sequence (5’-3’) |
| --- | --- | --- |
| hsa-miR-654-5p-mimic | S | UGGUGGGCCGCAGAACAUGUGC |
|  | AS | ACAUGUUCUGCGGCCCACCAUUU |
| hsa-miR-552-3p-mimic | S | AACAGGUGACUGGUUAGACAA |
|  | AS | GUCUAACCAGUCACCUGUUUU |
| hsa-miR-541-3p-mimic | S | UGGUGGGCACAGAAUCUGGACU |
|  | AS | UCCAGAUUCUGUGCCCACCAUUU |
| hsa-miR-NC | S | UUCUCCGAACGUGUCACGUTT |
|  | AS | ACGUGACACGUUCGGAGAATT |
| siHSPB1#1 | S | CCGAUGAGACUGCCGCCAAGUTT |
|  | AS | ACUUGGCGGCAGUCUCAUCGGTT |
| siHSPB1#2 | S | CCCAAGUUUCCUCCUCCCUGUTT |
|  | AS | U ACAGGGAGGAGGAAACUUGGGTT |
| siHSPB1#3 | S | GAUCACCAUCCCAGUCACCUUTT |
|  | AS | AAGGUGACUGGGAUGGUGAUCTT |
| siNC | S | UUCUCCGAACGUGUCACGUTT |
|  | AS | ACGUGACACGUUCGGAGAATT |
