# Supplementary material for: Engineered small extracellular vesicles loaded with miR-654-5p promote ferroptosis by targeting HSPB1 to alleviate sorafenib resistance in hepatocellular carcinoma
Source: Cell Death Discov. 2023 Sep 30;9:362. doi: 10.1038/s41420-023-01660-2 (PMC10542782; doi:10.1038/s41420-023-01660-2)
Supplement: Supplementary file 2 — Supplementary figure and figure legends [file 41420_2023_1660_MOESM2_ESM.docx]

**Supplementary figure and figure legends**

**
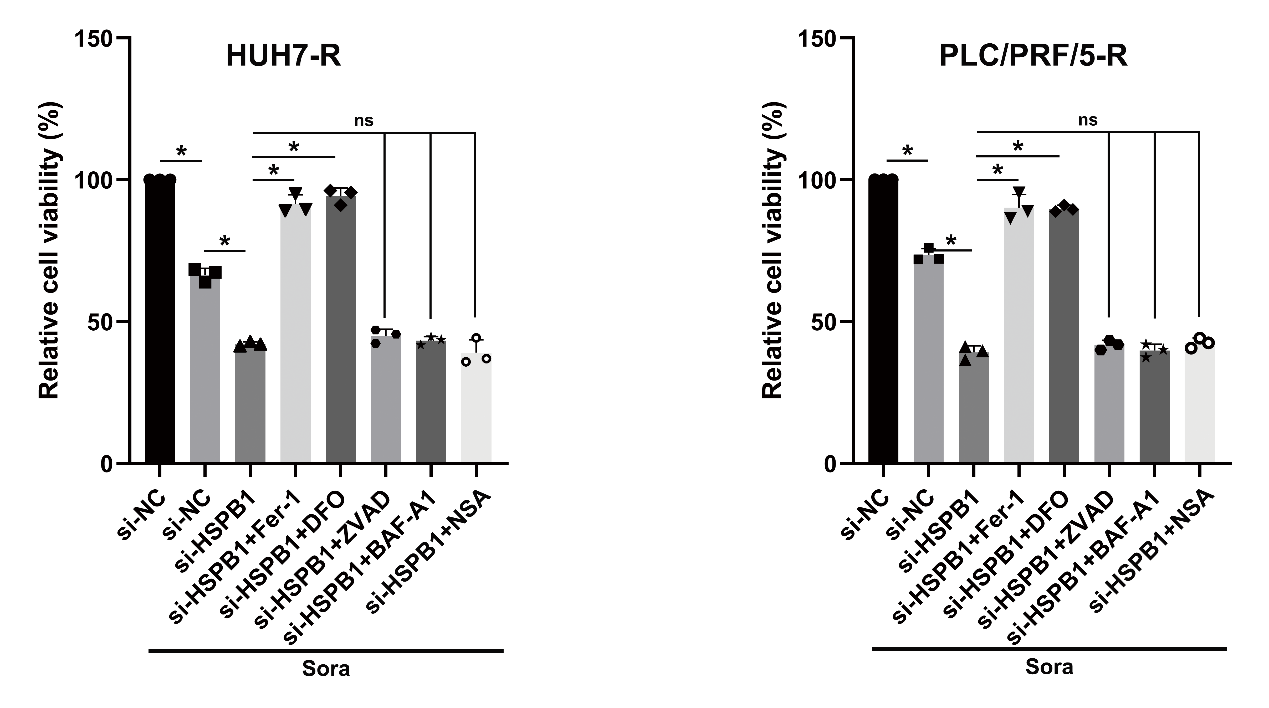
**

**Fig. S1 Knockdown of HSPB1 enhances sora-induced cell ferroptosis in HCC-R cells.** Cell viability of si-HSPB1 HCC-R cells treated with Sora (10 μM) alone or in combination with fer-1 (1 μM), DFO (100 μM), ZVAD (50 μM), BAF-A1 (50 μM), or NSA (2 μM) for 24 h. n=3 per group. Data are shown as the means ± SD, **p* < 0.05. ns: not significant. Fer-1: Ferrostatin-1. DFO: Deferoxamine. ZVAD: Z-VAD-FMK. BAF-A1: Bafilomycin. NSA: Necrosulfonamide.

**
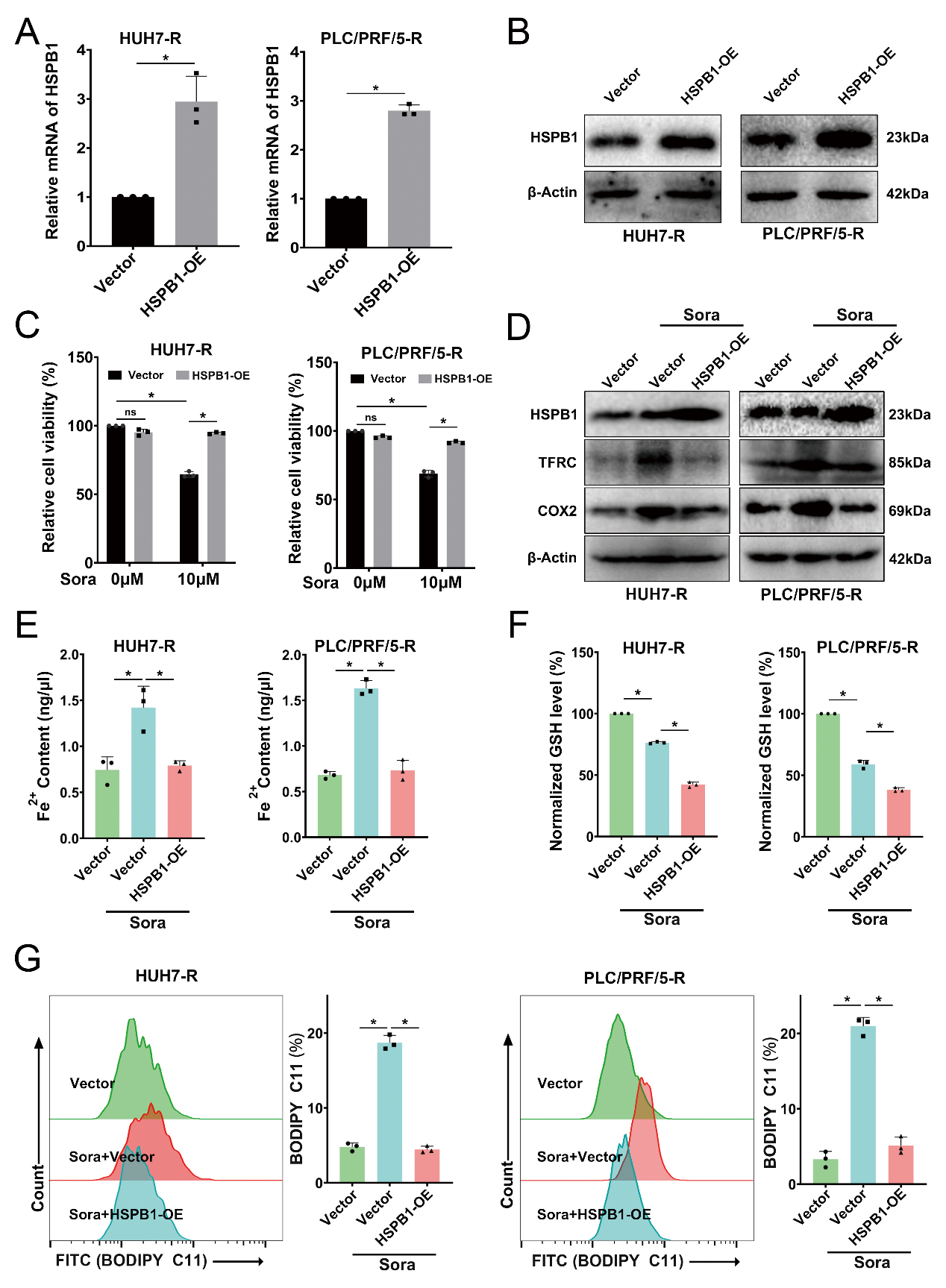
**

**Fig. S2 Overexpression of HSPB1 reverses sora-induced ferroptosis in HCC-R cells.** **(A, B)** HSPB1 mRNA **(A)** and protein **(B)** levels in HSPB1-OE cells. **(C)** Cell viability of HSPB1-OE cells treated with Sora. **(D)** WB analysis of HSPB1, TFRC, and COX2 protein changes in each group treated with Sora in HSPB1-OE cells, with β-Actin as an internal reference. **(E, F)** Fe2+ contents **(E)** and GSH level **(F)**. **(G)** Flow cytometry analysis of lipid ROS levels. n=3 per group. Data are shown as the means ± SD, **p*<0.05. ns: not significant.


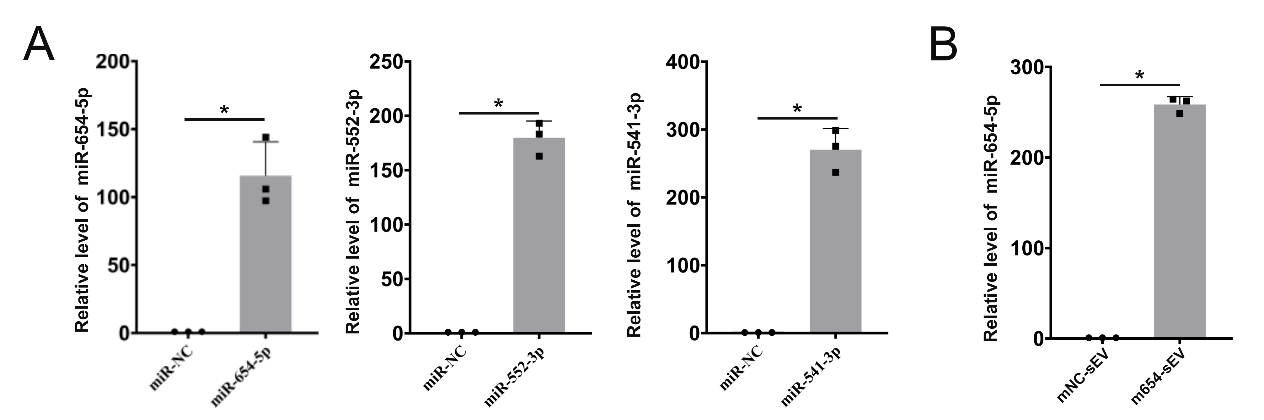


**Fig. S3** **Efficiency of transfection and loading of miRNA.** **(A)** The relative level of miR-654-5p, miR-552-3p, and miR-541-3p after transfection with miRNA-mimics. **(B)**The relative level of miR-654-5p in mNC-Exo and m654-exo. n=3 per group. Data are shown as the means ± SD, **p*<0.05. ns: not significant.

**
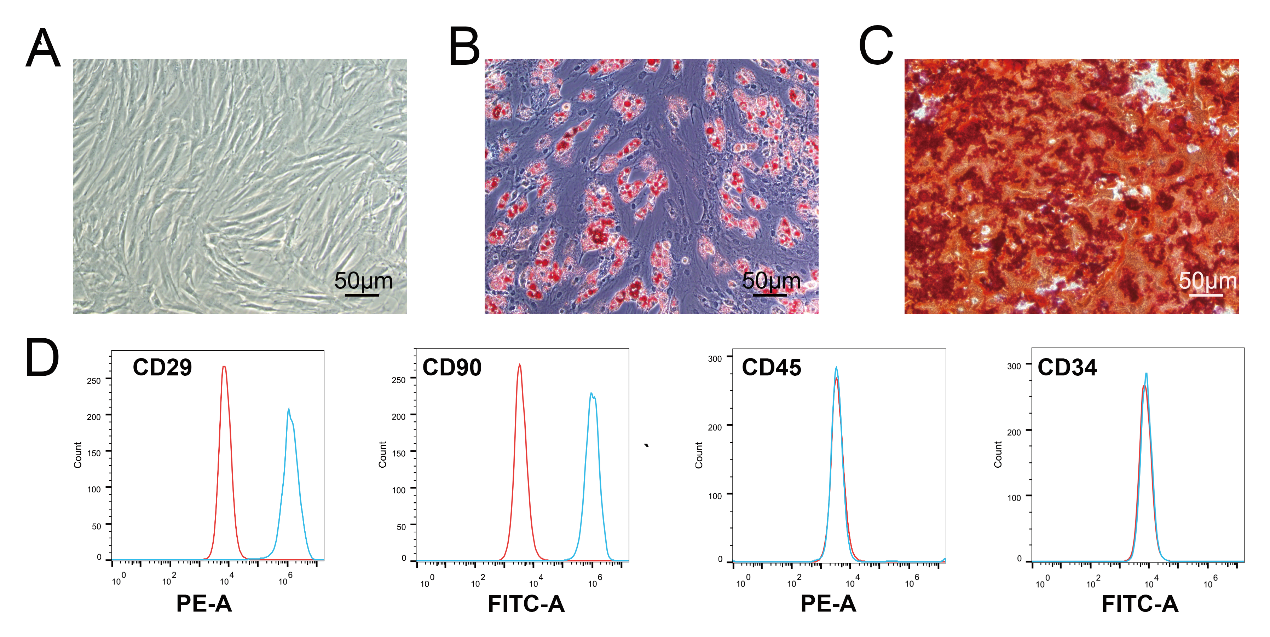
**

**Fig. S4 Characterization of human ADSCs**

**(A)** Morphology of stem cells under bright field. **(B, C)** ADSCs induced to differentiate into adipogenic **(B)** and osteogenic **(C)** lineages. **(D)** Flow cytometry identification of stem cell surface markers. Data are shown as the means ± SD, **p*<0.05. ns: not significant.
