# Supplementary material for: Engineered small extracellular vesicles loaded with miR-654-5p promote ferroptosis by targeting HSPB1 to alleviate sorafenib resistance in hepatocellular carcinoma
Source: Cell Death Discov. 2023 Sep 30;9:362. doi: 10.1038/s41420-023-01660-2 (PMC10542782; doi:10.1038/s41420-023-01660-2)
Supplement: Supplementary file 3 — Original western blots [file 41420_2023_1660_MOESM3_ESM.pdf]

Fig. 2F

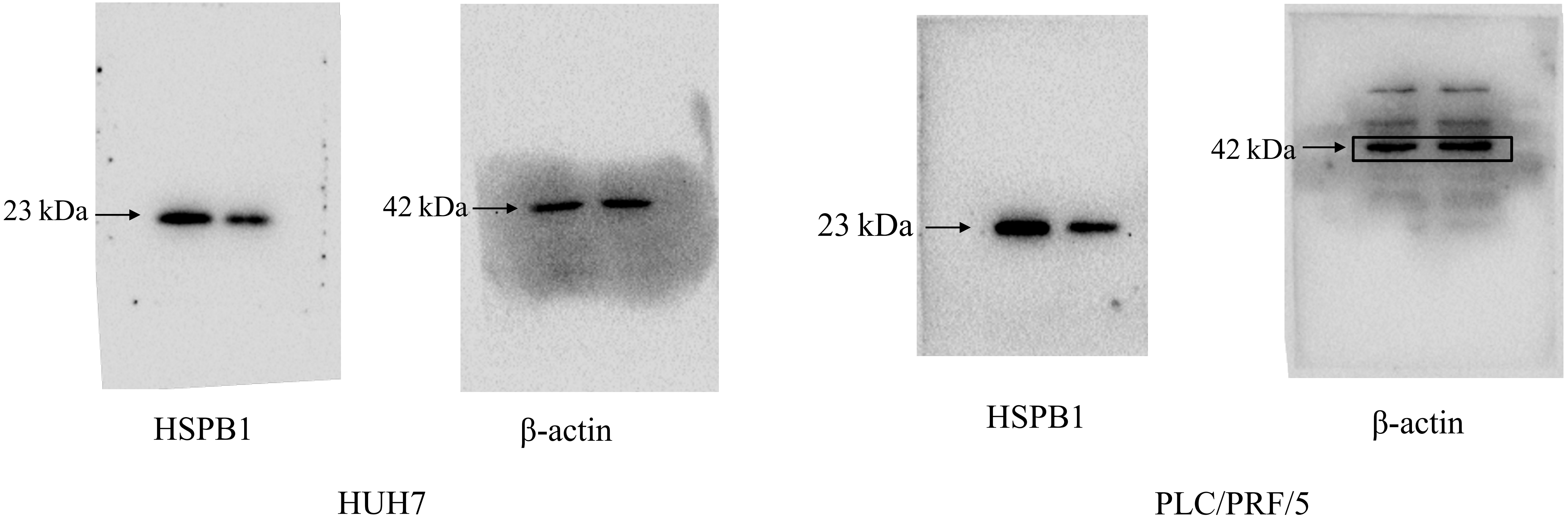
23 kDa
HSPB1
42 kDa
β-actin
HUH7
23 kDa
HSPB1
42 kDa
β-actin
PLC/PRF/5

Fig. 3B

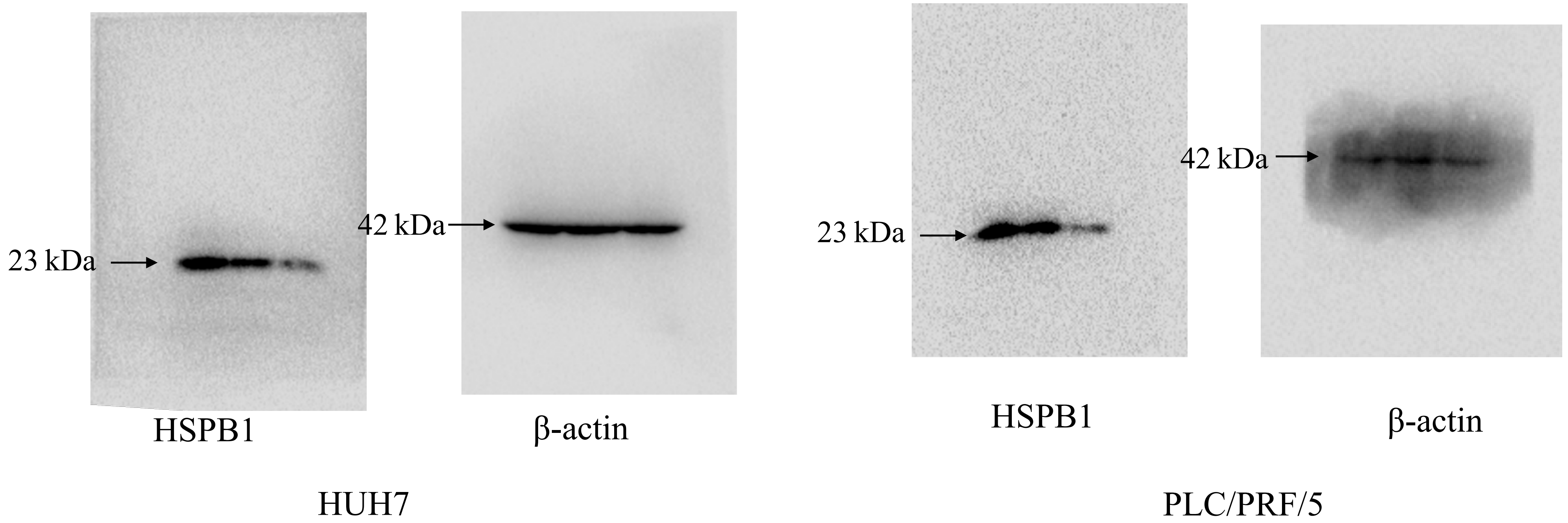
23 kDa
42 kDa
HSPB1
β-actin
HUH7
23 kDa
42 kDa
HSPB1
β-actin
PLC/PRF/5

Fig. 3E

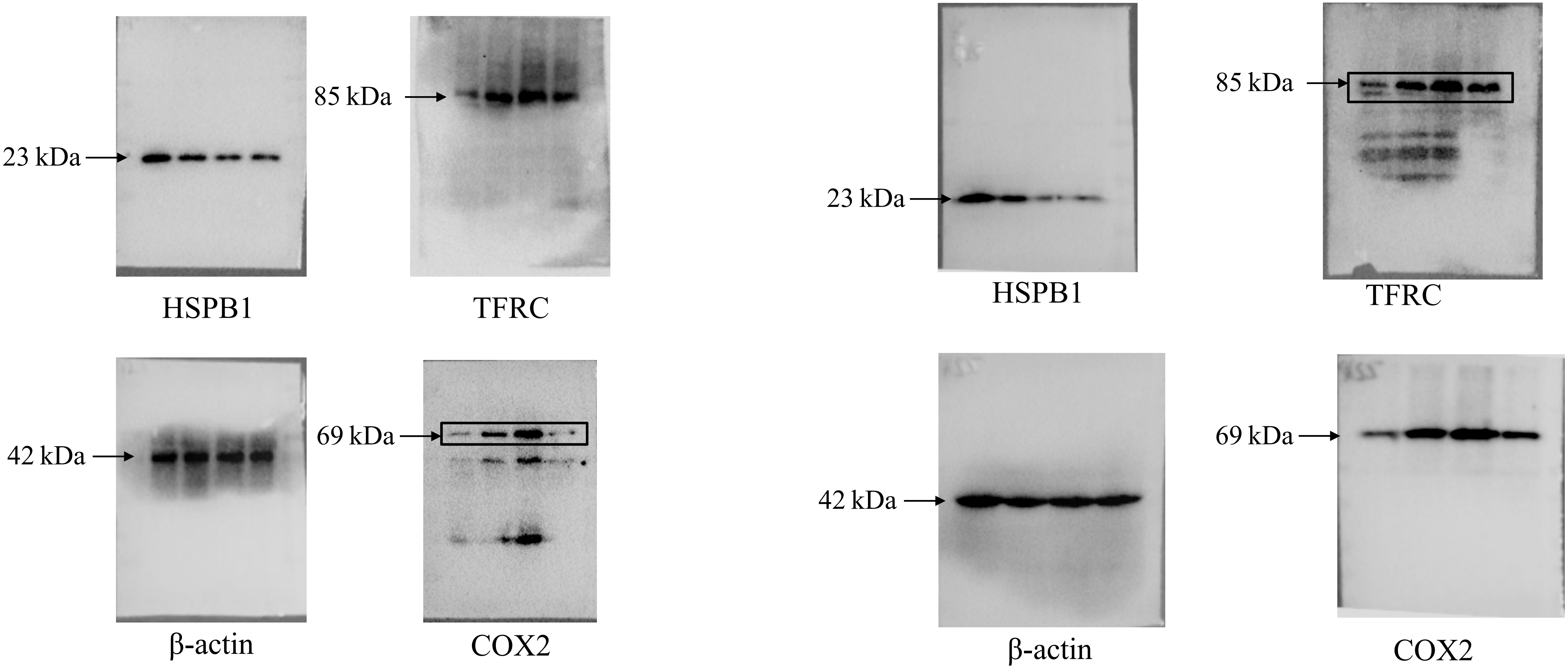

23 kDa
85 kDa
HSPB1
TFRC
42 kDa
69 kDa
β-actin
COX2
85 kDa
23 kDa
HSPB1
TFRC
42 kDa
69 kDa
β-actin
COX2

Fig. 4C

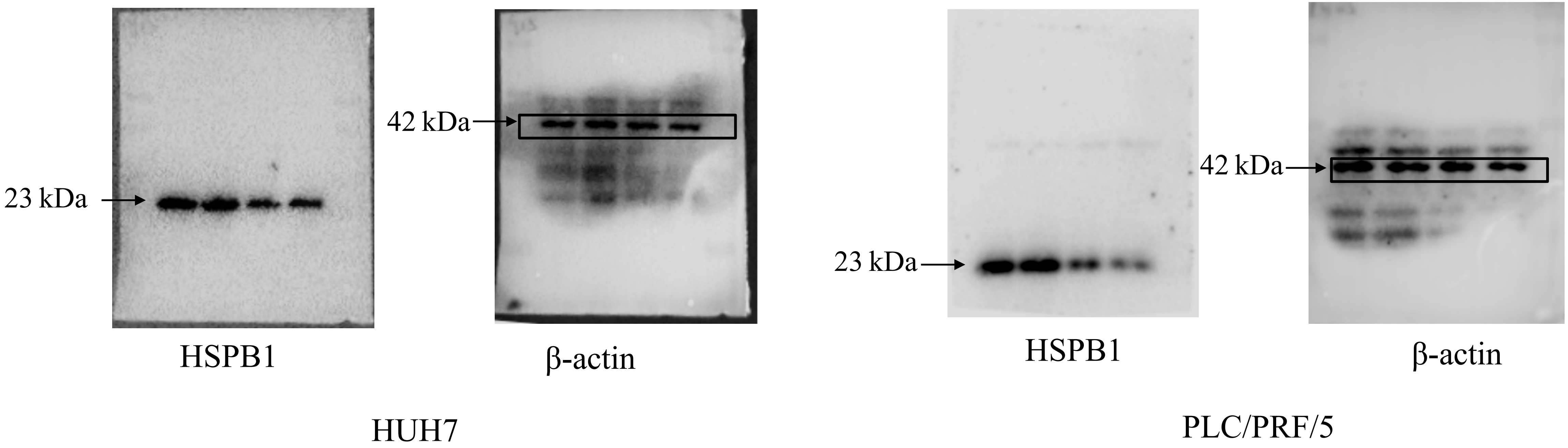
23 kDa
42 kDa
HSPB1
β-actin
HUH7
42 kDa
23 kDa
HSPB1
β-actin
PLC/PRF/5

Fig. 4G

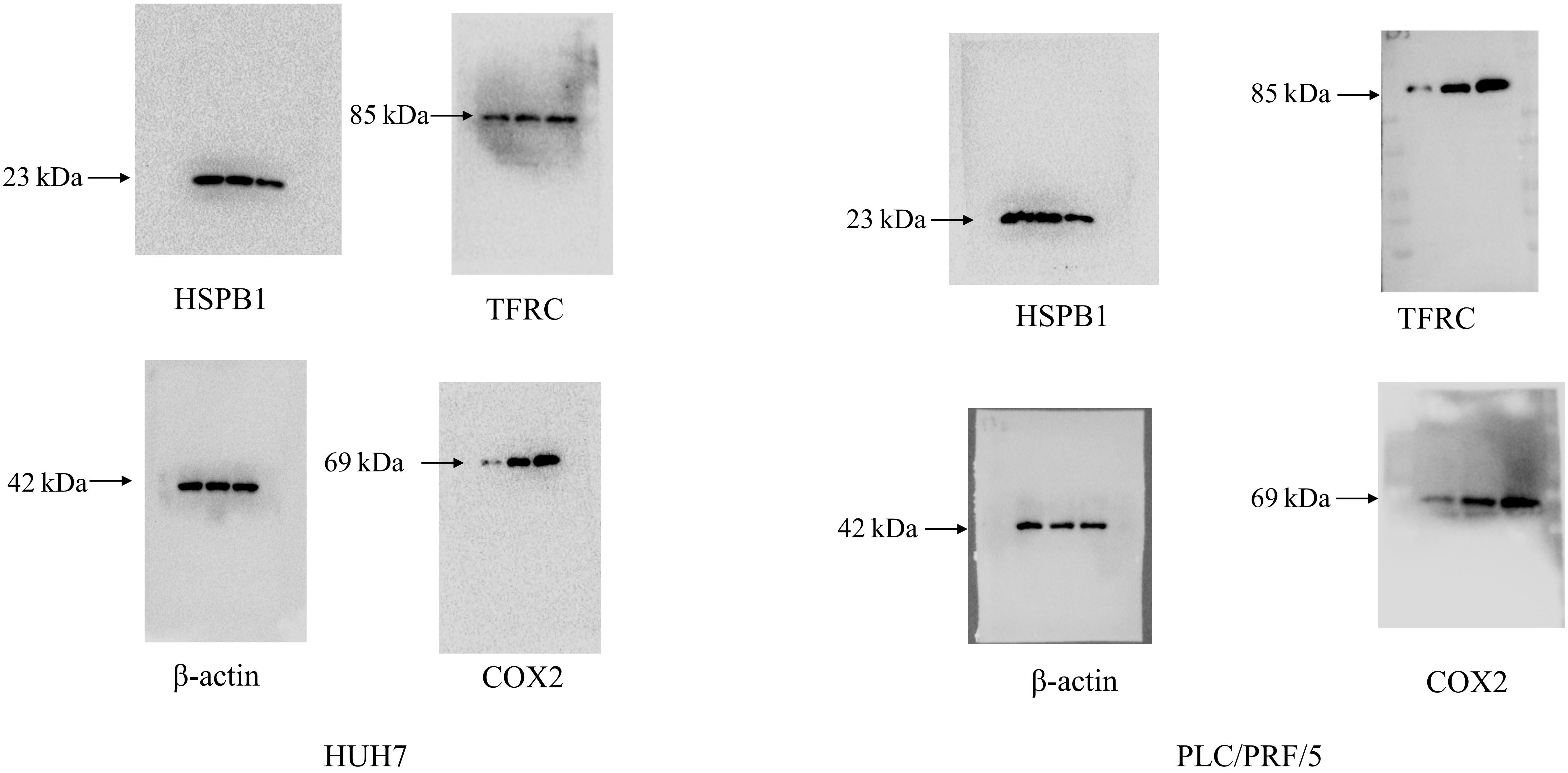
23 kDa
85 kDa
HSPB1
TFRC
42 kDa
69 kDa
β-actin
COX2
HUH7
23 kDa
85 kDa
HSPB1
TFRC
42 kDa
69 kDa
β-actin
COX2
PLC/PRF/5

Fig. 5B

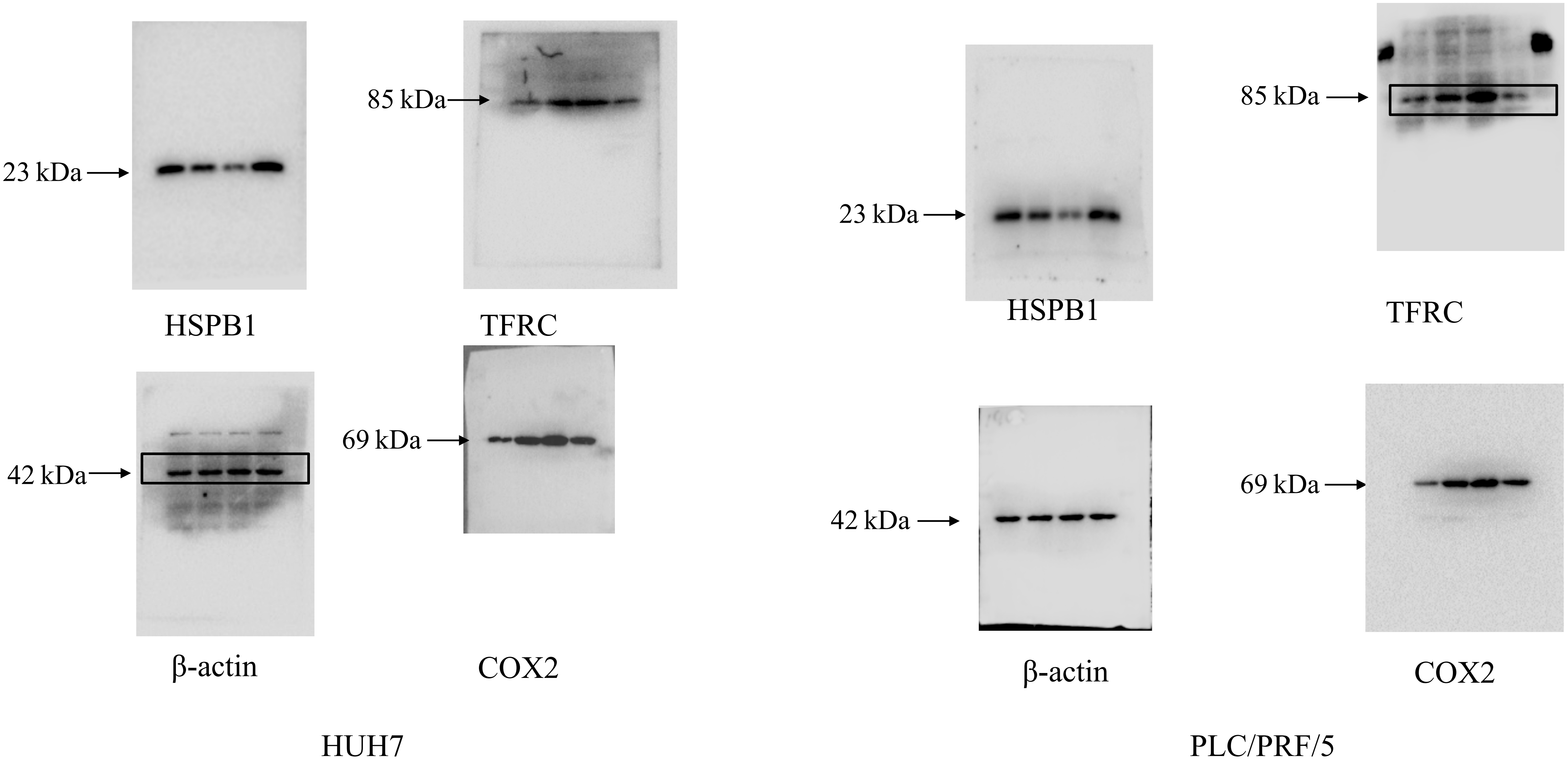
23 kDa
HSPB1
85 kDa
TFRC
42 kDa
β-actin
69 kDa
COX2
HUH7
23 kDa
HSPB1
85 kDa
TFRC
42 kDa
β-actin
69 kDa
COX2
PLC/PRF/5

Fig. 6C

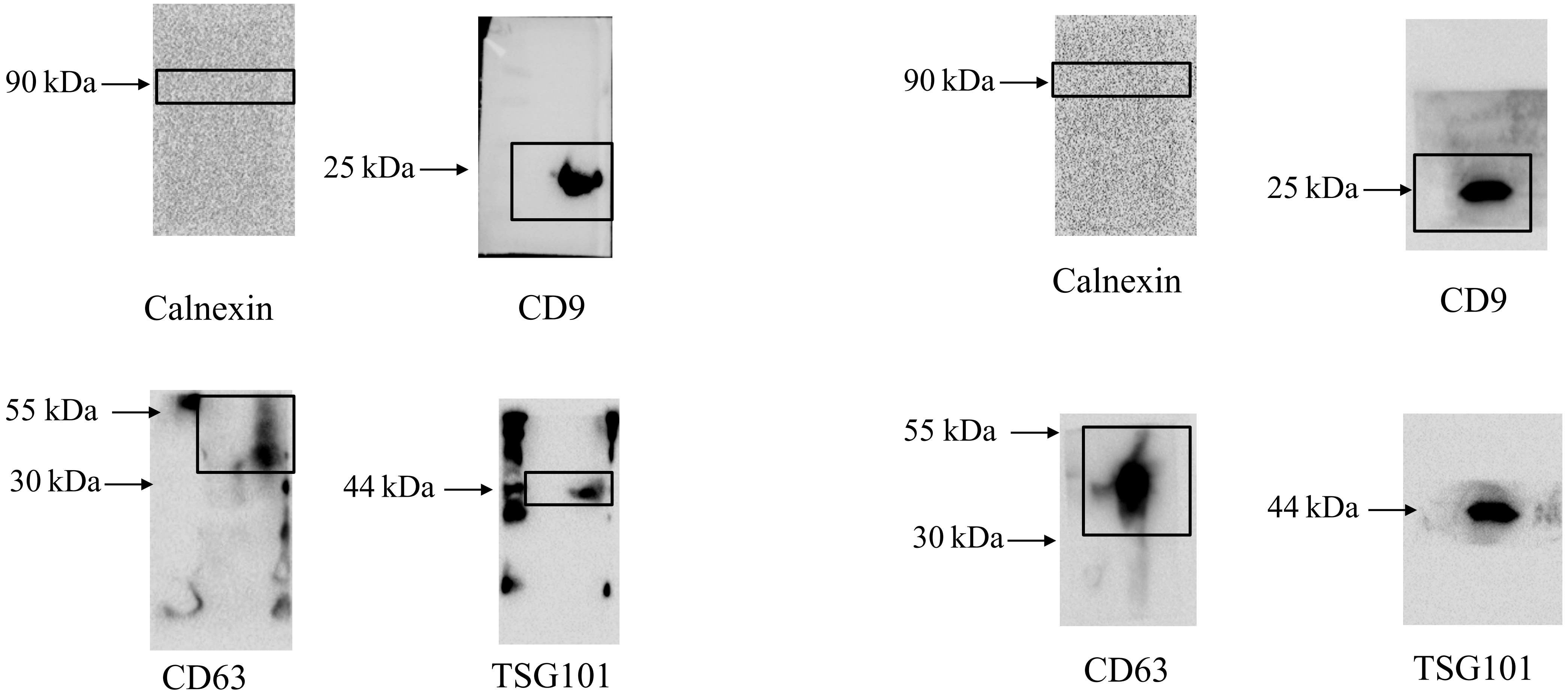
90 kDa
Calnexin
25 kDa
CD9
55 kDa
30 kDa
CD63
44 kDa
TSG101
90 kDa
Calnexin
25 kDa
CD9
55 kDa
30 kDa
CD63
44 kDa
TSG101

Fig. 6I

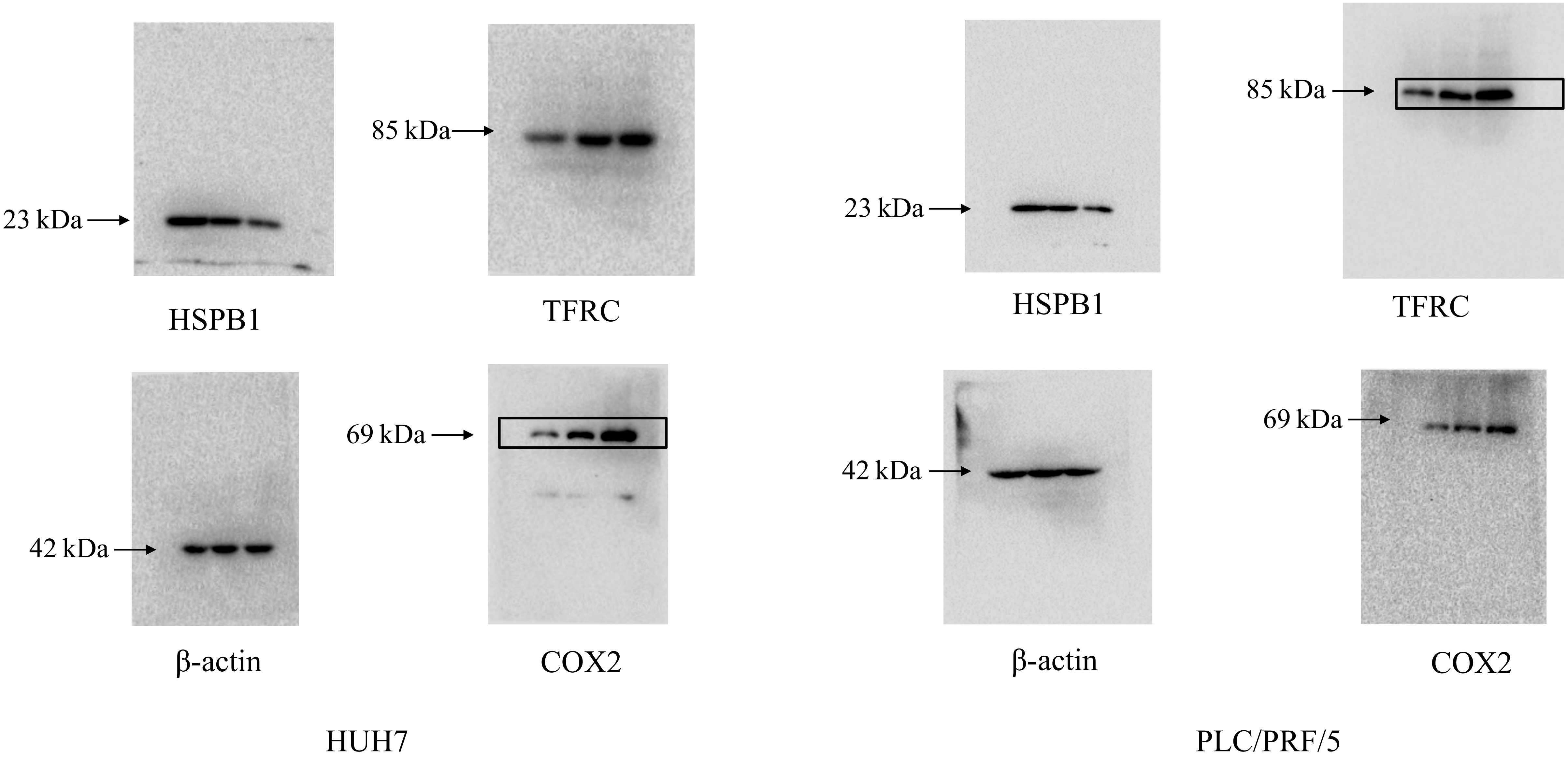
23 kDa
HSPB1
85 kDa
TFRC
42 kDa
β-actin
69 kDa
COX2
HUH7
23 kDa
HSPB1
85 kDa
TFRC
42 kDa
β-actin
69 kDa
COX2
PLC/PRF/5

Fig. 7F

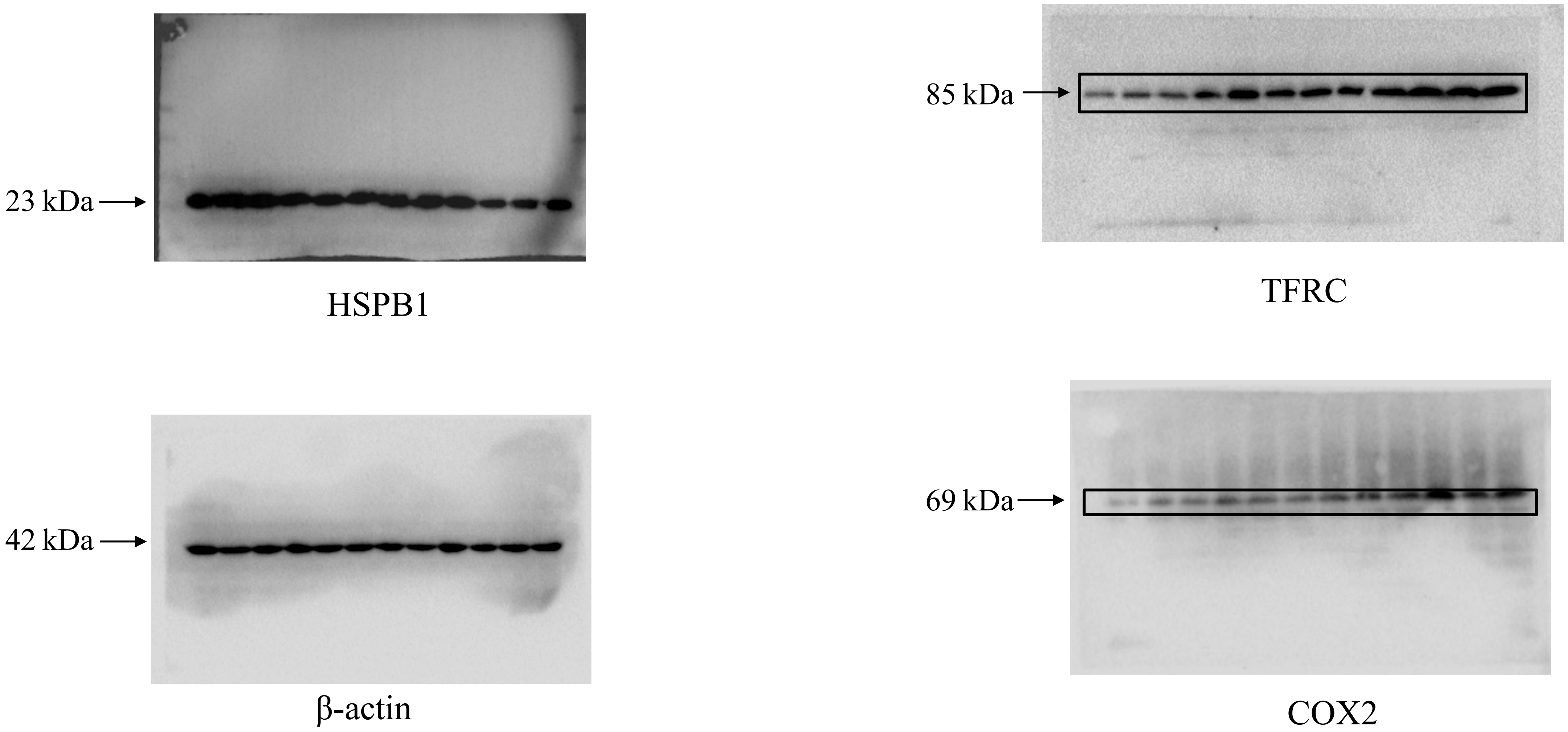

Fig. S2B

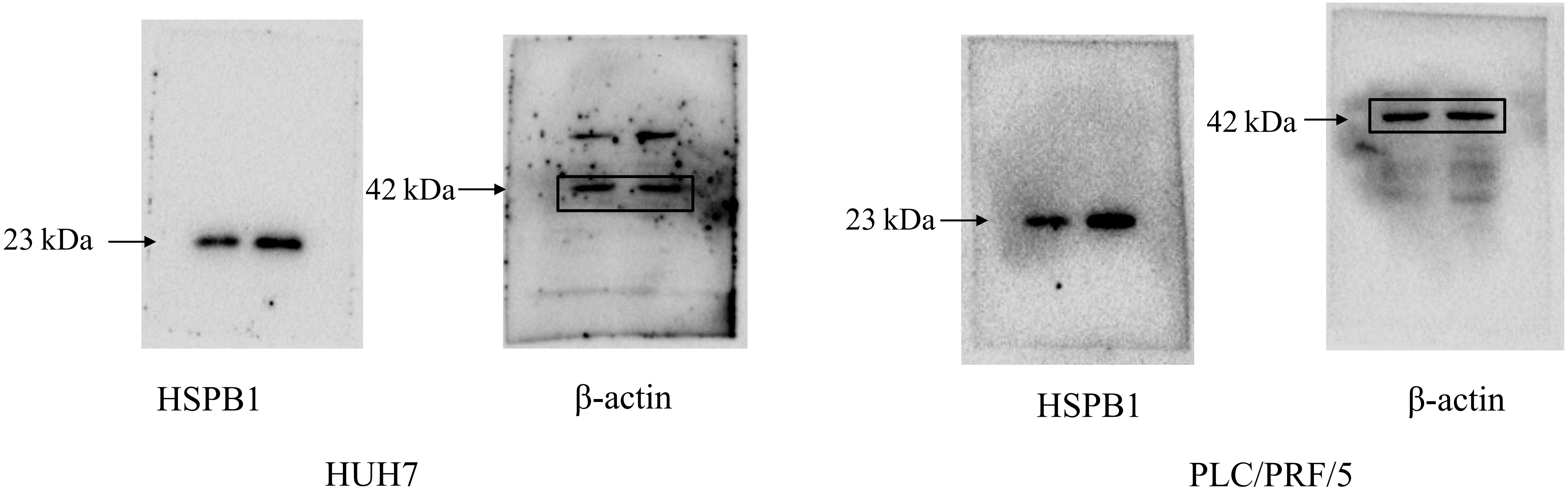
23 kDa
42 kDa
HSPB1
β-actin
HUH7
23 kDa
42 kDa
HSPB1
β-actin
PLC/PRF/5

Fig. S2D

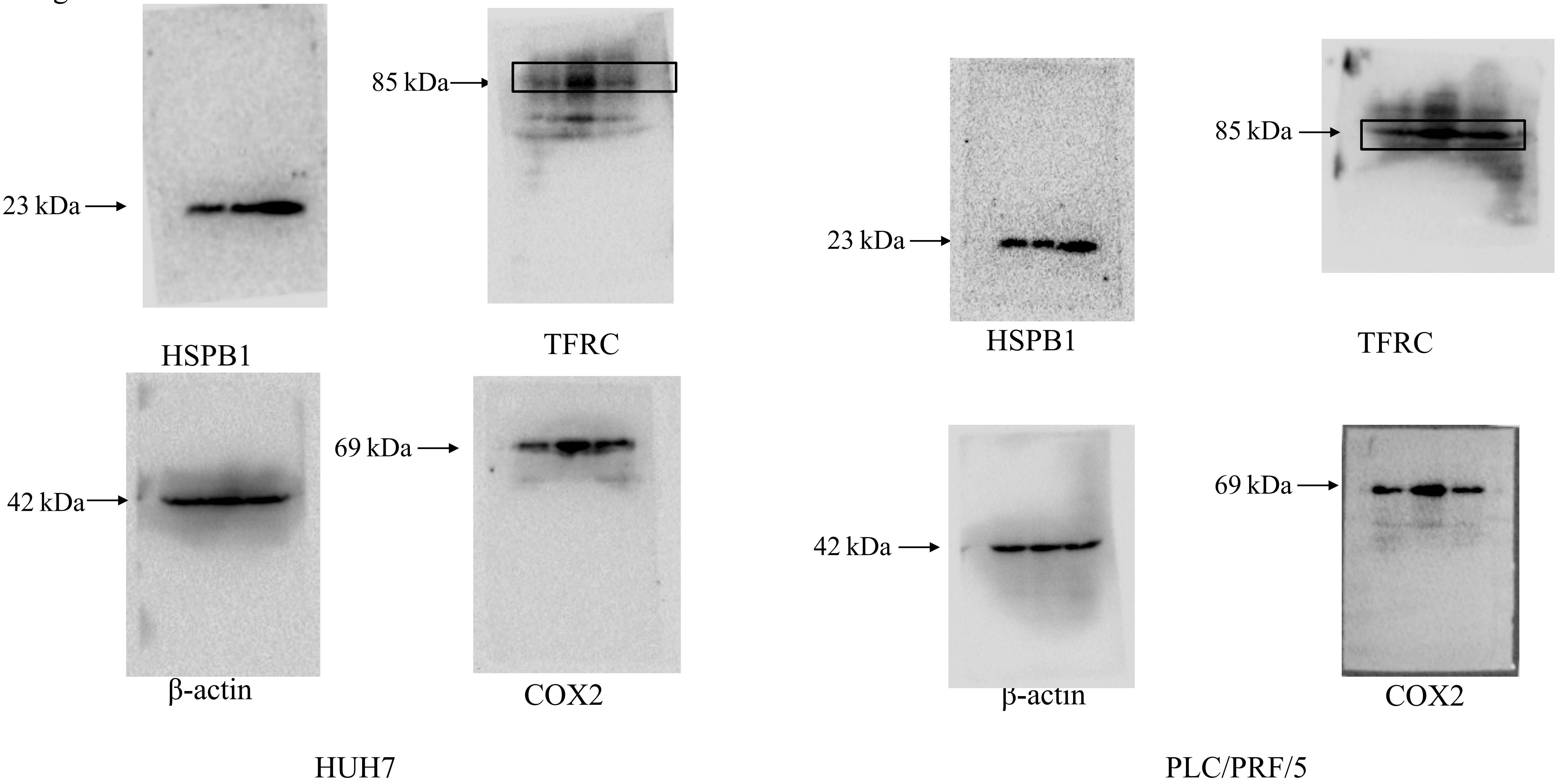
23 kDa
85 kDa
HSPB1
TFRC
42 kDa
69 kDa
β-actin
COX2
HUH7
23 kDa
85 kDa
HSPB1
TFRC
42 kDa
69 kDa
β-actin
COX2
PLC/PRF/5
